# Ti/CuO Nanothermite—Study of the Combustion Process

**DOI:** 10.3390/molecules29163932

**Published:** 2024-08-20

**Authors:** Mateusz Polis, Konrad Szydło, Barbara Lisiecka, Marcin Procek, Tomasz Gołofit, Tomasz Jarosz, Łukasz Hawełek, Agnieszka Stolarczyk

**Affiliations:** 1 Łukasiewicz Research Network—Explosive Techniques Research Group, Institute of Industrial Organic Chemistry, 42-693 Krupski Młyn, Poland; 2 Department of Physical Chemistry and Technology of Polymers, Silesian University of Technology, 44-100 Gliwice, Poland; 3 Department of Optoelectronics, Silesian University of Technology, 2 Krzywoustego Str., 44-100 Gliwice, Poland; 4 Faculty of Chemistry, Warsaw University of Technology, Noakowskiego 3, 00-664 Warsaw, Poland; 5 Lukasiewicz Research Network—Institute of Non-Ferrous Metals, 5 Sowinskiego St., 44-100 Gliwice, Poland

**Keywords:** nanothermite, energetic material, combustion

## Abstract

A study of the combustion processes of Ti/CuO and Ti/CuO/NC nanothermites prepared via electrospraying was conducted in this work. For this purpose, the compositions were thermally conditioned at 350, 550 and 750 °C, as selected based on our initial differential scanning calorimetry-thermogravimetry (DSC/TG) investigations. The tested compositions were analysed for chemical composition and morphology using SEM-EDS, Raman spectroscopy and XRD measurements. Additionally, the thermal behaviour and decomposition kinetics of compositions were explored by means of DSC/TG. The Kissinger and Ozawa methods were applied to the DSC curves to calculate the reaction activation energy. SEM-EDS analyses indicated that sintering accelerated with increasing equivalence ratio and there was a strong effect on the sintering process due to cellulose nitrate (NC) addition. The main combustion reaction was found to start at 420–450 °C, as confirmed by XRD and Raman study of samples annealed at 350 °C and 550 °C. Moreover, increasing the fuel content in the composition led to lower E_a_, higher reaction heats and a more violent combustion process. Conversely, the addition of NC had an ambiguous effect on E_a_. Finally, a multi-step combustion mechanism was proposed and is to some extent in line with the more general reactive sintering (RS) mechanism. However, unusual mass transfer was observed, i.e., to the fuel core, rather than the opposite, which is typically observed for Al-based nanothermites.

## 1. Introduction

Nanothermites (NTs), which originally were referred to as metastable interstitial composites [1], are one of the newest classes of pyrotechnic systems. NTs have found a broad array of prospective applications, both civilian and military in nature. Among potential applications of NT systems, their use in the construction of igniters, microthrusters, primers, gas generating systems and safety switches [2,3,4] is expected. Moreover, NTs may find use in biocidal applications, welding and in the construction of detectors [5,6,7]. This significant practical potential has brought about many research efforts into NTs, encompassing their combustion mechanisms, optimising their performance and designing novel NT systems [8,9,10,11].

Among the various NT formulations reported in the literature, most reports are dedicated to Al/CuO [12], which is a nigh-model system. Similarly, many works have been dedicated to Al-based NTs, such as Al/NiFe_2_O_4_ [13], Al/WO_3_ [14] and Al/Mn_2_O_3_ [15]. The variety of oxidising agents used for NTs is not matched by the variety of fuels, with Al-based NTs being the most commonly reported in the literature. Recently, however, Ti was reported as a potential alternative to Al [16].

Although the Ti/CuO system has been studied both theoretically and practically [17], virtually no information is available on the mechanism of its combustion and on the impact of gas-generating additives on this mechanism. Tests have been performed to investigate the effect of Ti nanopowder addition to Al-based thermites [18,19] and limited studies have been conducted on the Ti/CuO system [20].

The lack of an established mechanistic description of the combustion of titanium-based thermites may lead to the flawed assumption that their combustion takes place along the same route as the much more comprehensively described aluminothermites. To exemplify, for aluminothermites, the formation of an aluminium oxide shell on the fuel grains rapidly becomes irrelevant during the evolution of combustion due ot the low melting point of Al. Conversely, for Ti-based (melting point of 1650 K) nanothermites, even during the main combustion stage, temperatures may not be sufficient to liquefy the entirety of the fuel, greatly increasing the influence of the Ti oxide shell on shaping the reactivity of the fuel.

Although macroscopic processes, such as the above-mentioned formation of and changes in the titanium oxide shell, cannot govern the occurring chemical reactions, they shape the kinetics of these reactions to a significant extent. Consequently, even an initial combustion mechanism needs to corroborate these processes with the occurrence of individual chemical reactions, so as to provide an accurate picture of the ongoing processes. This task is made even more complex by the inclusion of additives (e.g., cellulose nitrate (NC)) that significantly affect the macroscopic mass and heat transfer conditions in the combusting system. In this work, we have endeavoured to achieve such a mechanism, encompassing both the combustion of a “pure” Ti/CuO nanothermite, as well as the combustion of a NC-supplemented Ti/CuO nanthothermite.

## 2. Results

### 2.1. SEM-EDS Tests

#### 2.1.1. Raw Samples

**Figure A1:**
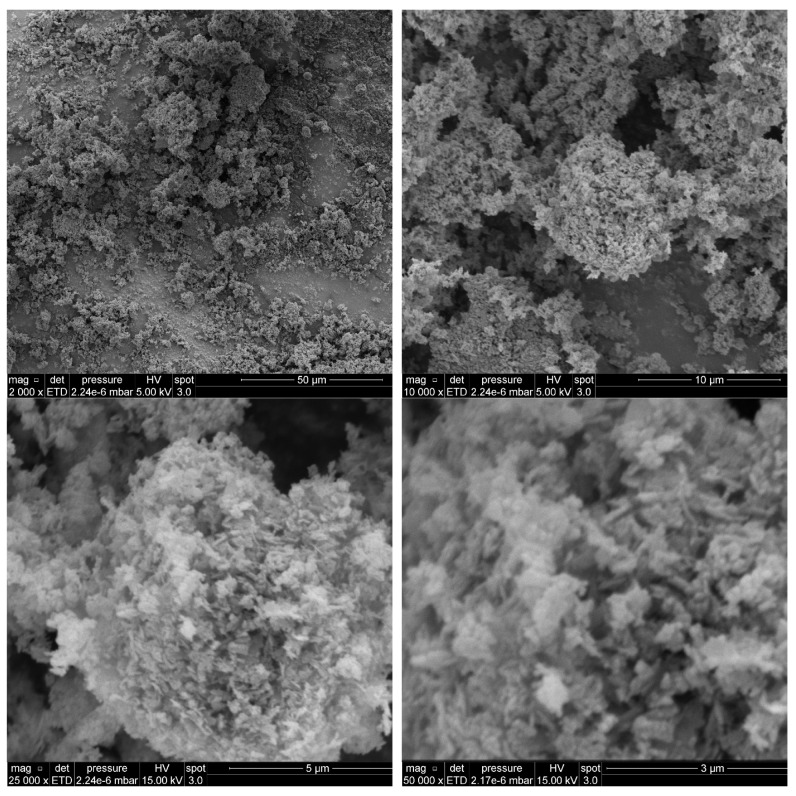
SEM images for C2 composition after preparation.

**Figure A2:**
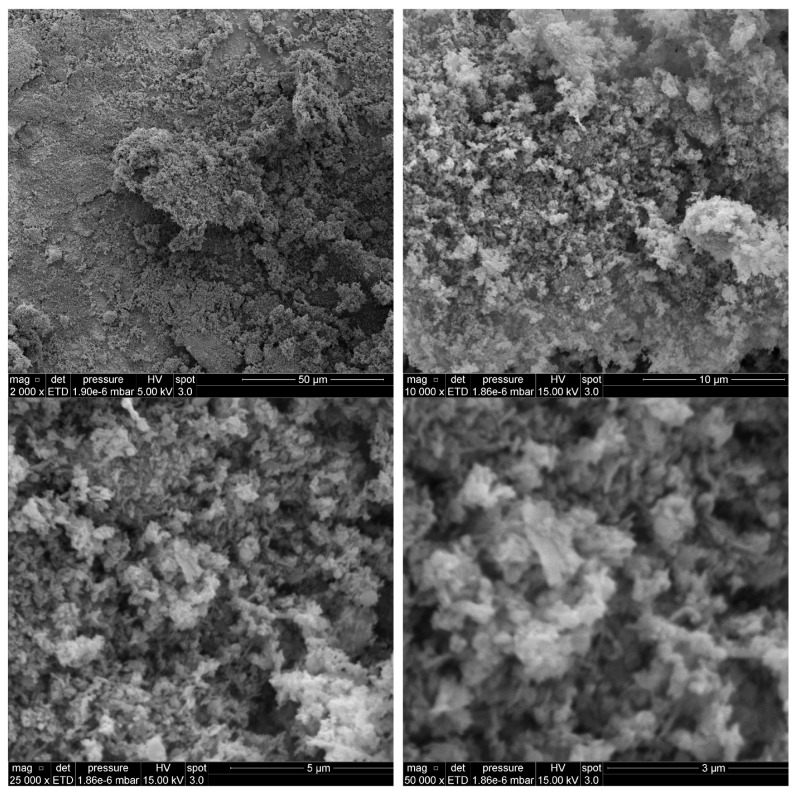
SEM images for C3 composition after preparation.

**Figure A3:**
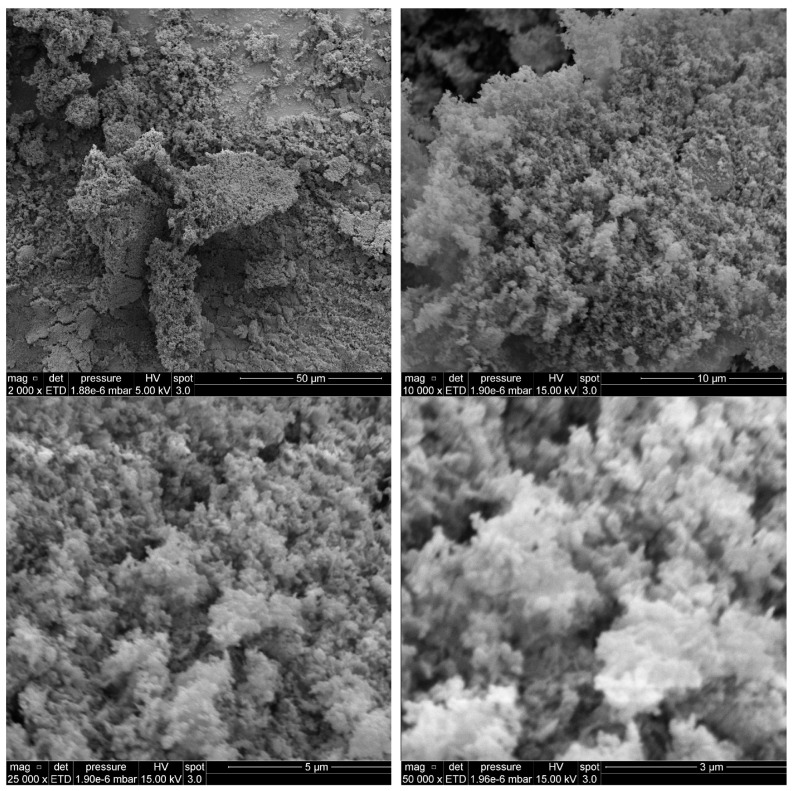
SEM images for C4 composition after preparation.

**Figure A4:**
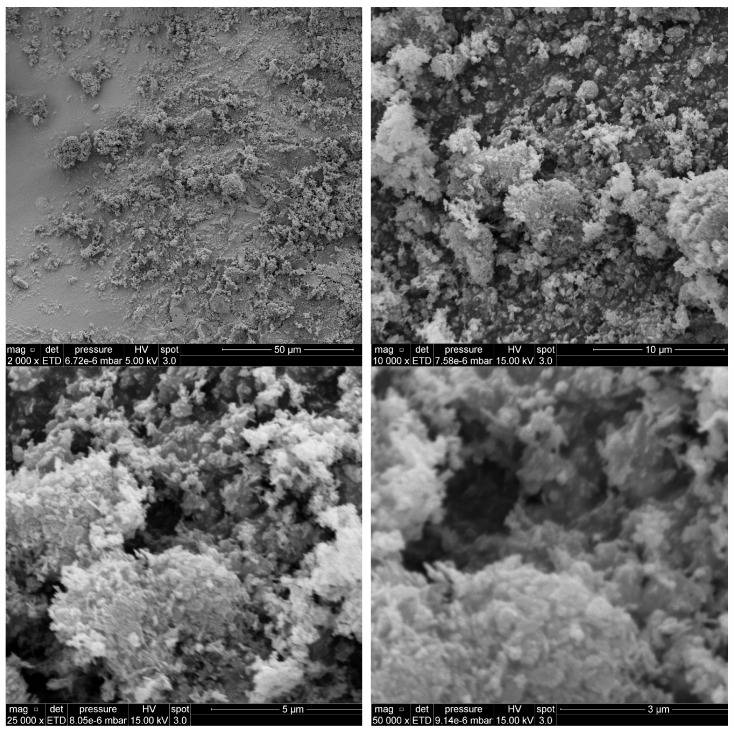
SEM images for C3-1NC composition after preparation.

SEM analyses of raw NT samples after their preparation confirms the nanometric size of the used components (Figure A1, Figure A2, Figure A3 and Figure A4). However, the presence of single micrometric grains of metallic fuels is also indicated. The compositions are composed mostly of agglomerates, composed of smaller granular and spherical particles, which results from their high surface energy. The addition of NC to the system yields larger but more homogeneous particles. Additionally, the presence of spindle-like particles may also be observed. SEM-EDS analyses confirmed relatively high homogenisation of the components (Figure A17, Figure A18, Figure A19 and Figure A20). Nevertheless, the presence of micrometric Ti particles and Ti tending to agglomerate into larger clusters may be identified, as well as an exclusion effect between Ti and Cu atoms.

#### 2.1.2. Thermal Conditioning of Compositions

**Figure A5:**
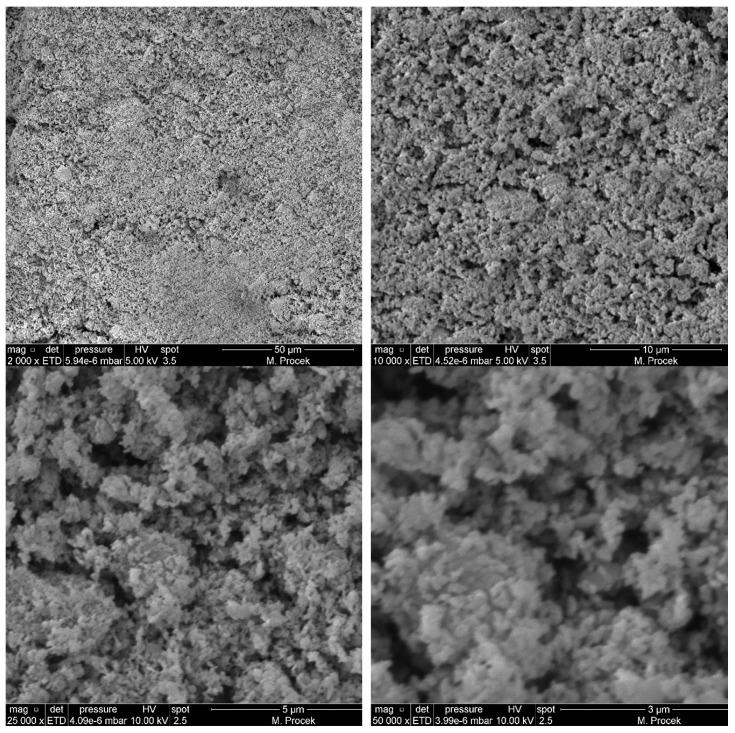
SEM images for C2 composition annealed at 350 °C.

**Figure A6:**
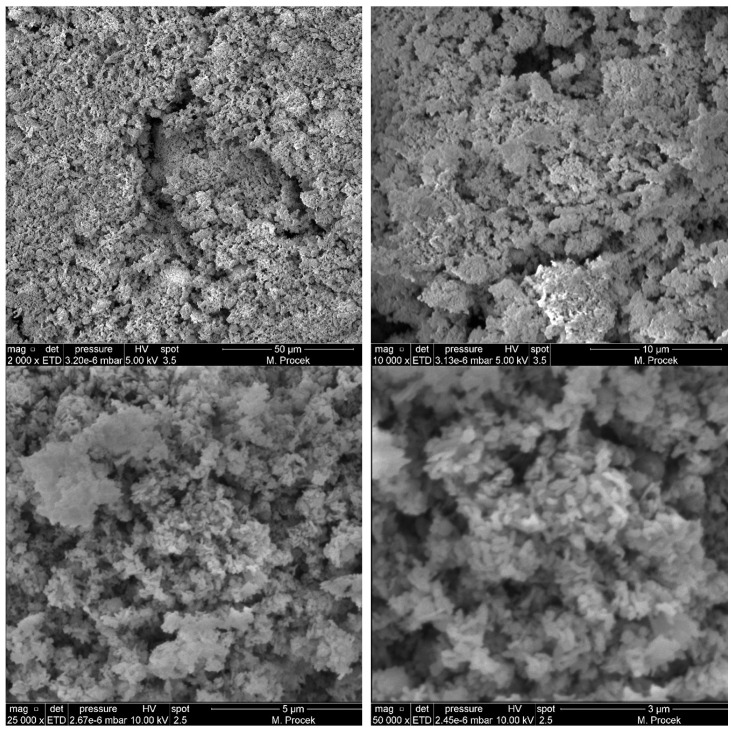
SEM images for C3 composition annealed at 350 °C.

SEM analysis of annealed samples is in line with other performed tests. For the C2 composition that was annealed at 350 °C (Figure A5), preliminary sintering of the system can be observed, resulting in the presence of agglomerates composed of spherical and spindle particles. Single, massive sintered particles covered with smaller, mostly spherical particles can be observed as well. The whole system is porous, with caverns being present. After annealing at 550 °C (Figure A9), the number of agglomerates increased rapidly; however, the spherical and granular microstructure of sintered and agglomerated particles is clearly visible. The sample that was annealed at 750 °C (Figure A13) is made up of large, sintered particles. The morphology of these particles is mostly multi-walled, whereas a small number of little grains with multi-walled or hemispherical morphology could be found. At the same time, the surface is quite smooth and non-porous. The C3 composition that was annealed at 350 °C (Figure A6) presents similar morphology to that of C2. However, the agglomerates are composed of flat, flower-like structures and the constituent grains are more spindle-like than spherical. After annealing at 550 °C, the morphology of the C3 composition became strongly sintered and porous (Figure A10). The large, multi-walled sintered particles are covered with fluffy, small grains with a wide spread of morphologies. Samples annealed at 750 °C (Figure A14) present strongly sintered morphology, similar to the C2 samples annealed at the same temperature, but a few cracks and caverns are present.

**Figure 1:**
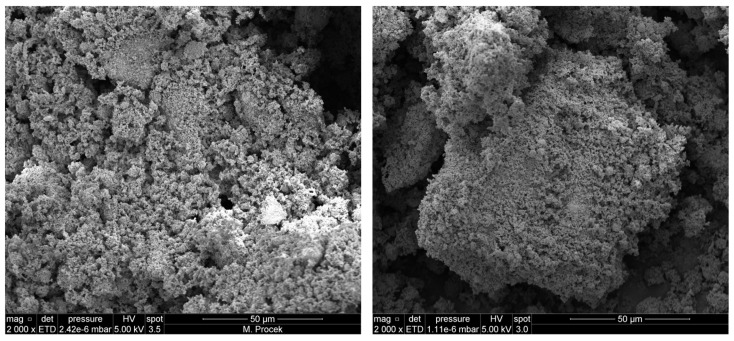
SEM images of the C3 (on the **left**) and C4 (on the **right**) compositions after annealing at 350 °C.

**Figure A7:**
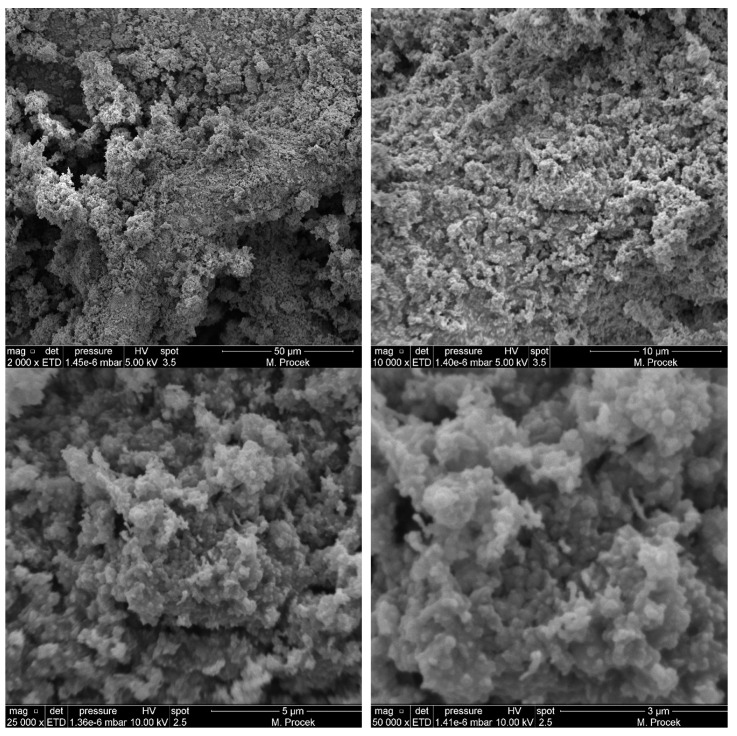
SEM images for C4 composition annealed at 350 °C.

**Figure A8:**
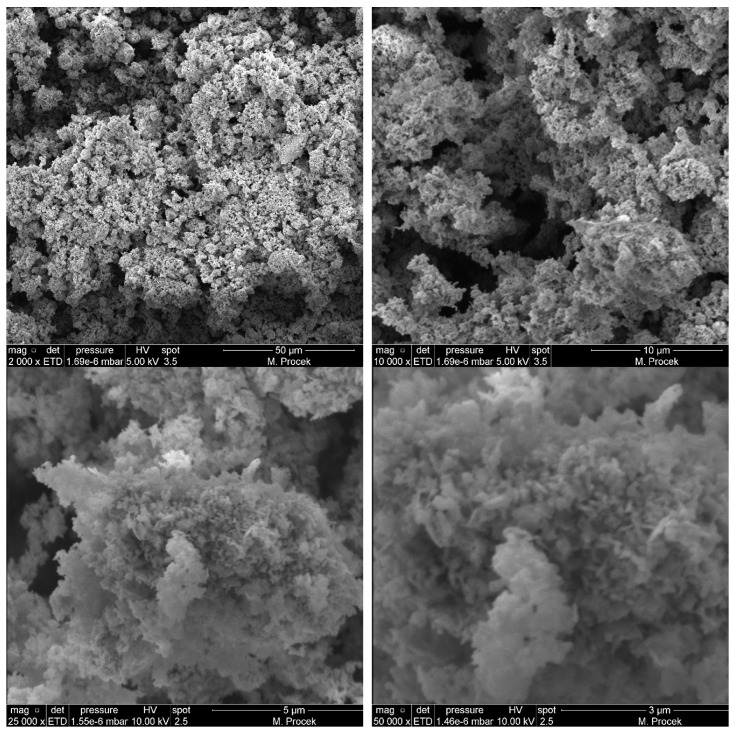
SEM images for C3-1NC composition annealed at 350 °C.

**Figure A9:**
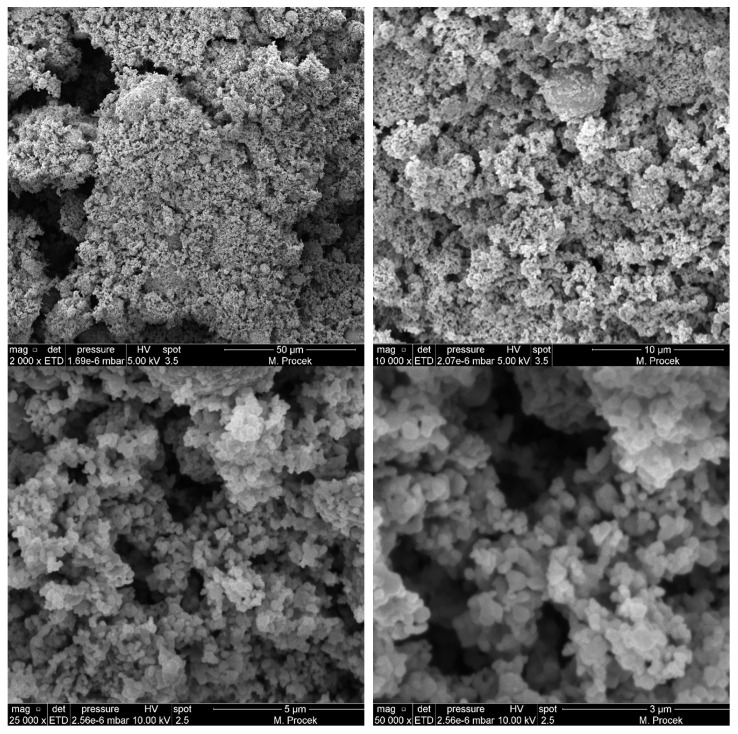
SEM images for C2 composition annealed at 550 °C.

**Figure A10:**
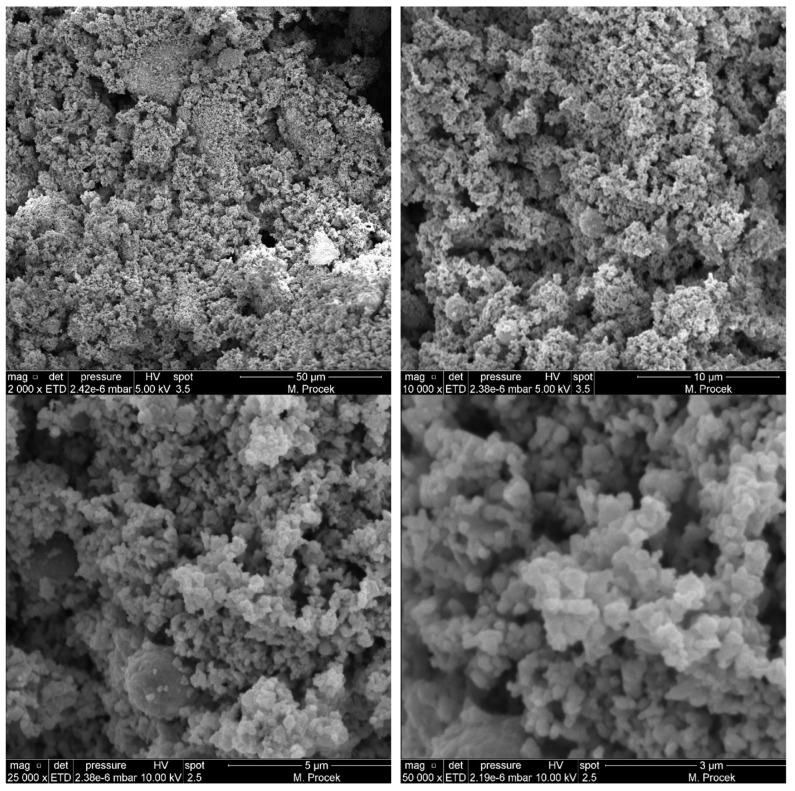
SEM images for C3 composition annealed at 550 °C.

**Figure A11:**
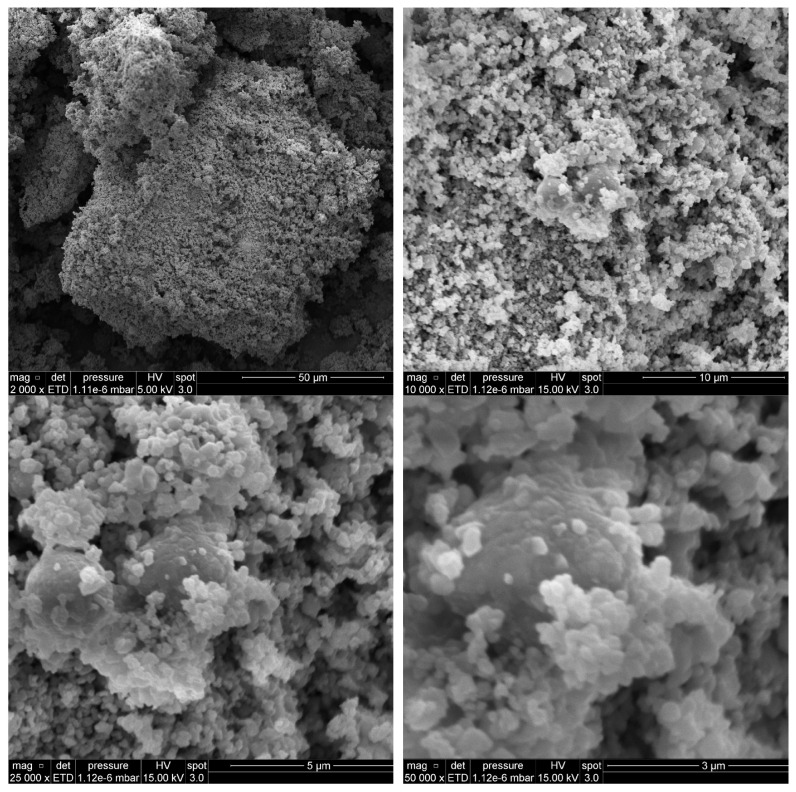
SEM images for C4 composition annealed at 550 °C.

**Figure A12:**
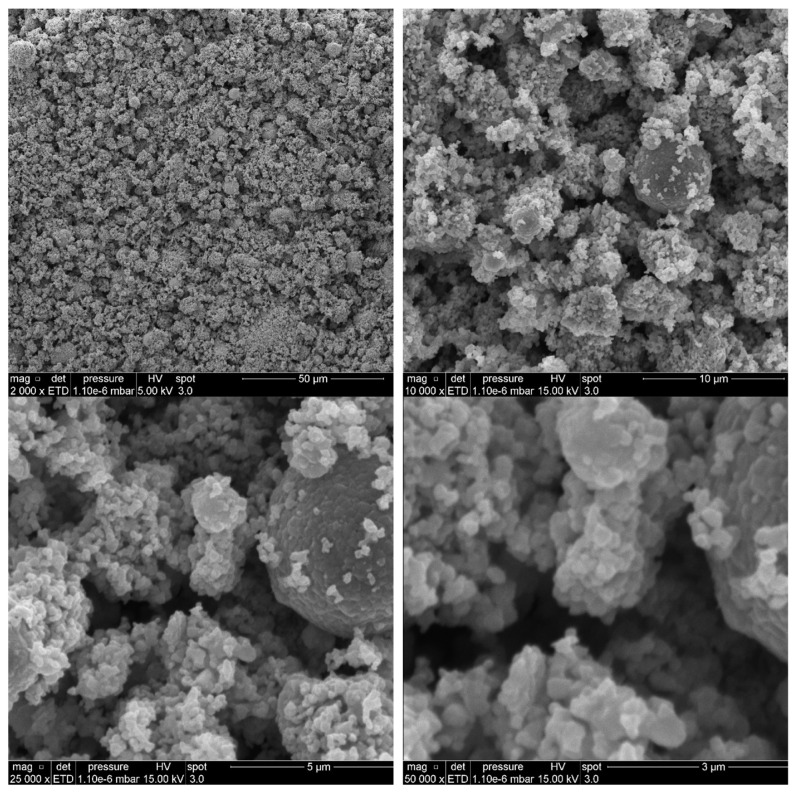
SEM images for C3-1NC composition annealed at 550 °C.

**Figure A13:**
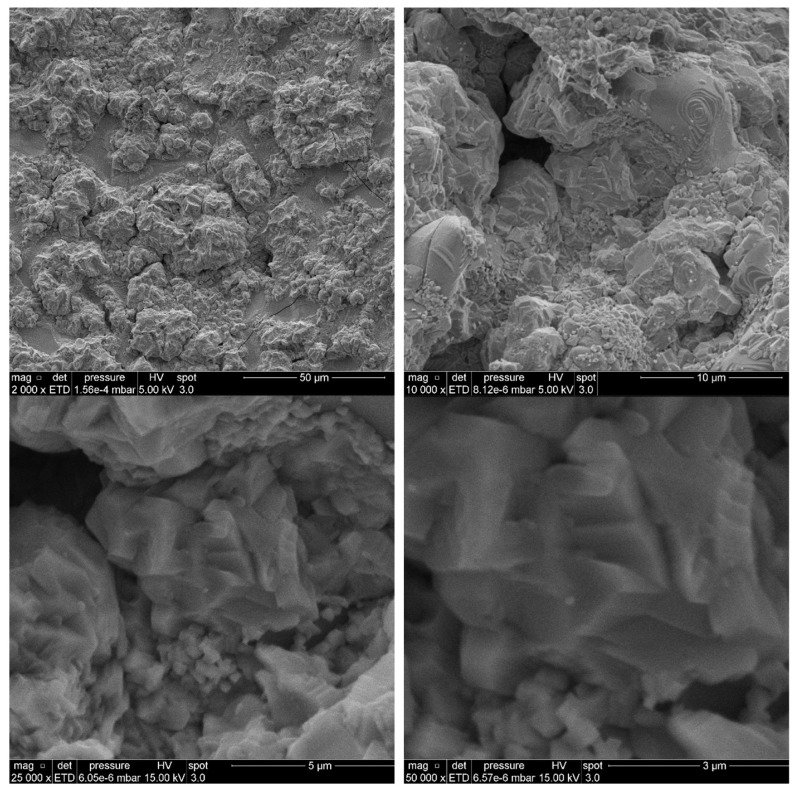
SEM images for C2 composition annealed at 750 °C.

**Figure A14:**
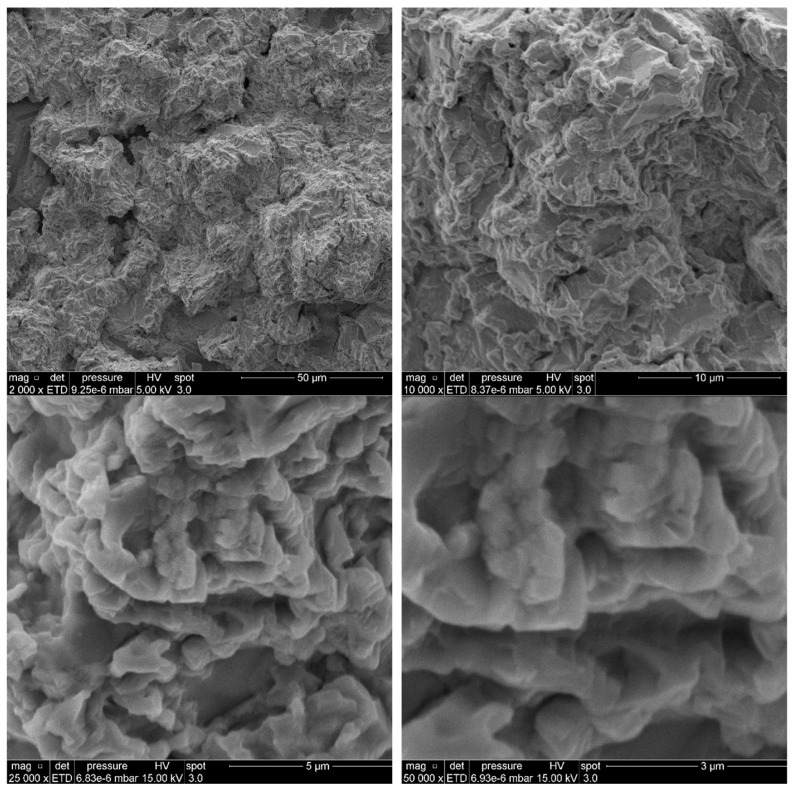
SEM images for C3 composition annealed at 750 °C.

**Figure A15:**
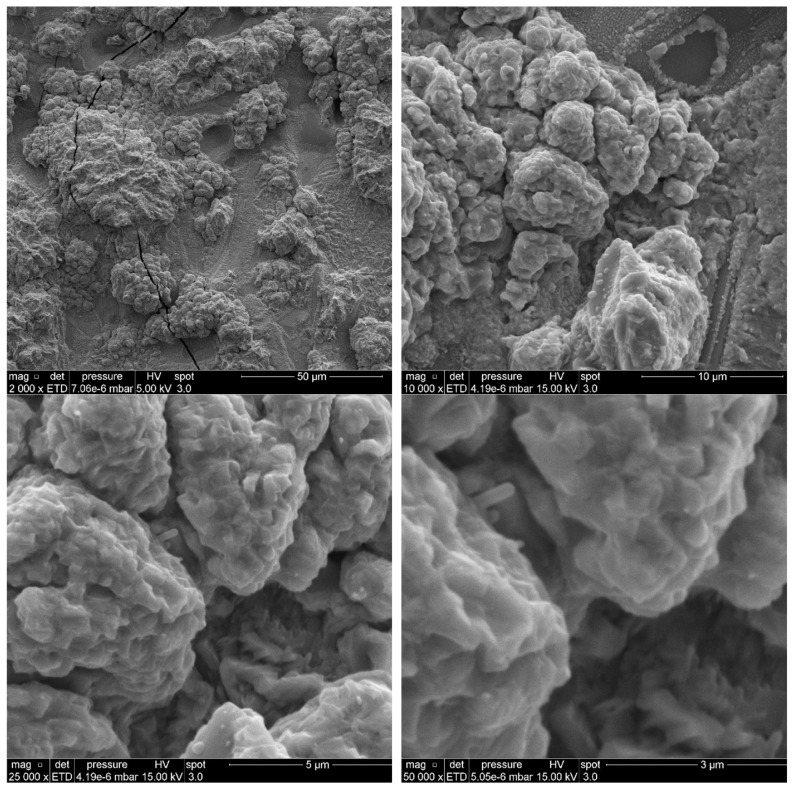
SEM images for C4 composition annealed at 750 °C.

**Figure A16:**
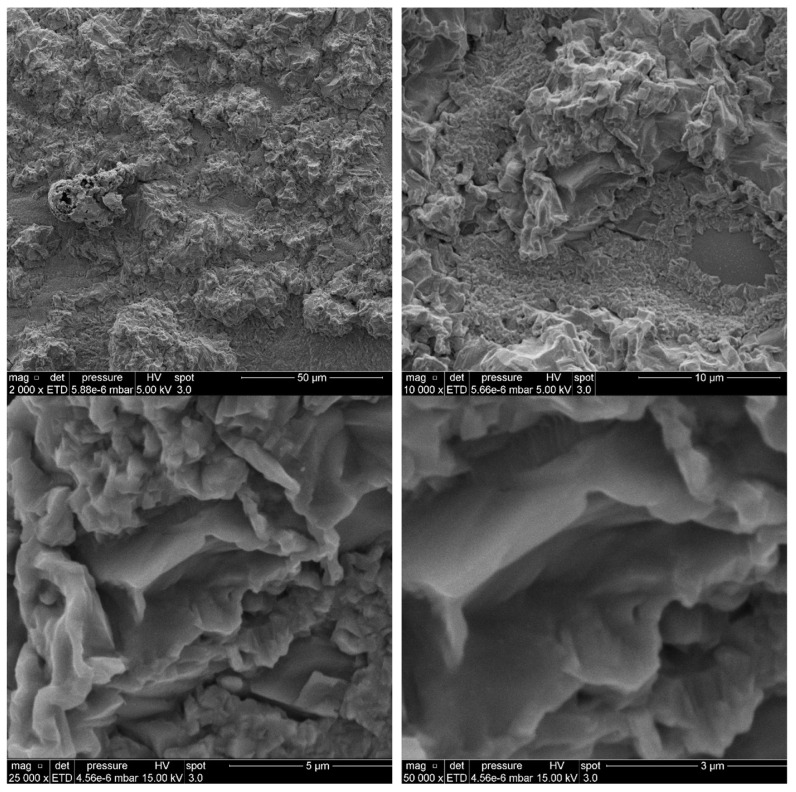
SEM images for C3-1NC composition annealed at 750 °C.

**Figure A17:**
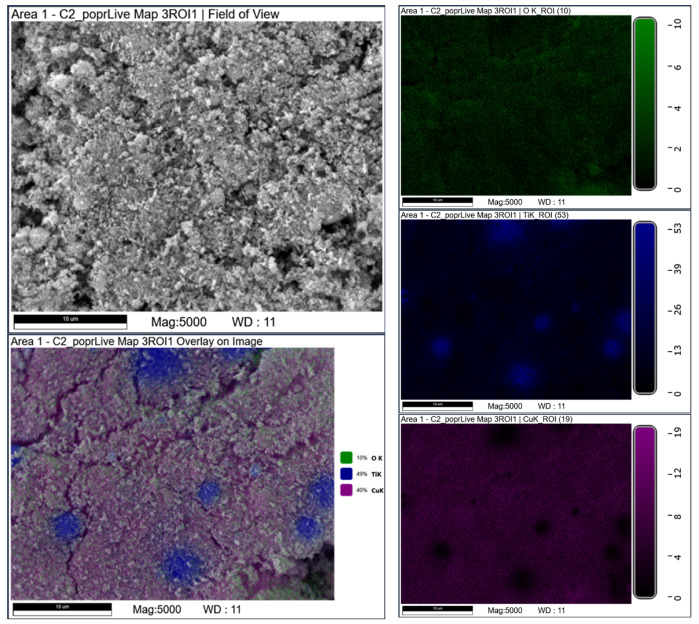
SEM-EDS images for C2 composition after preparation.

**Figure A18:**
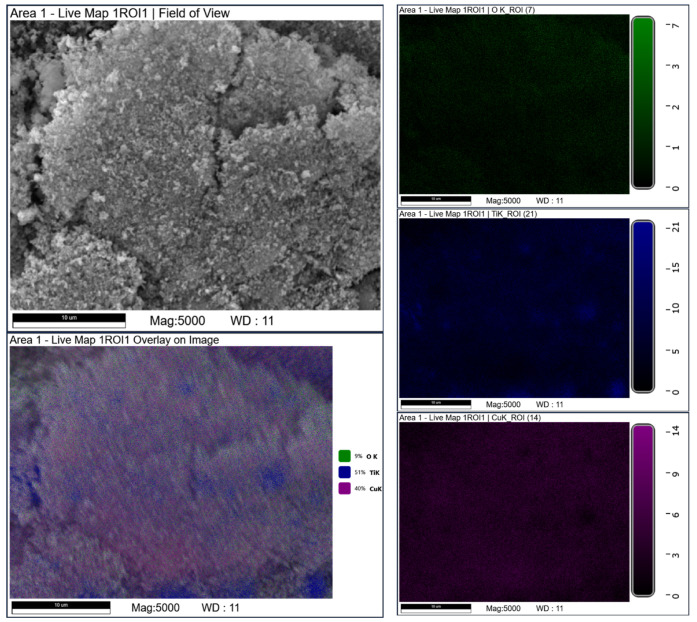
SEM-EDS images for C3 composition after preparation.

**Figure A19:**
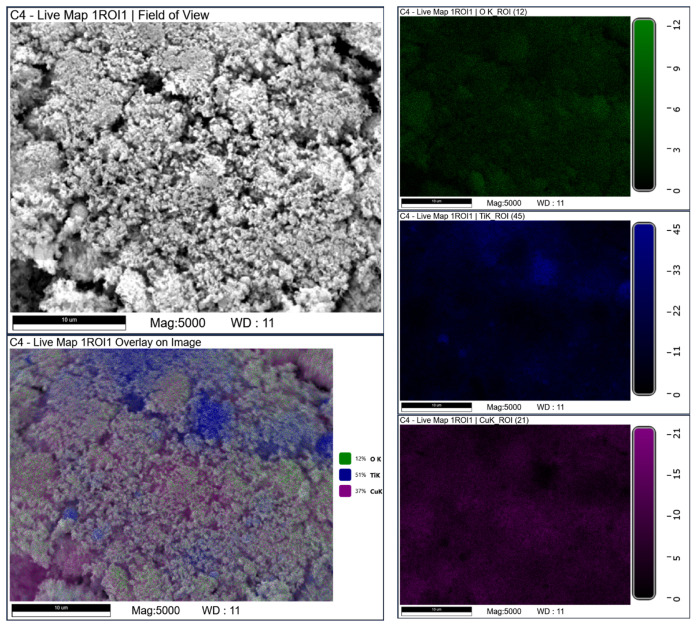
SEM-EDS images for C4 composition after preparation.

**Figure A20:**
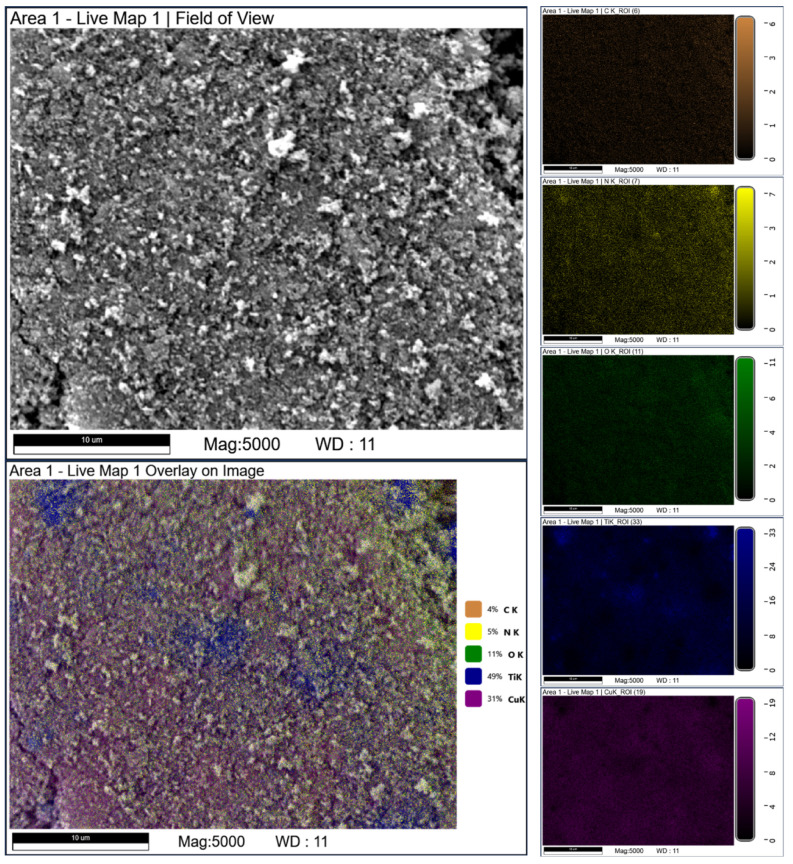
SEM-EDS images for C3-1NC composition after preparation.

In the case of the C4 composition, samples annealed at 350 °C (Figure A7) showed the presence of sintered, uneven, large particles covered with small fluffy grains. The whole structure is porous and is full of cracks and caverns—the structure resembles the structure of previous samples annealed at 550 °C—Figure 1. The C4-550 sample (Figure A11) is composed mostly of large sintered grains that are full of cracks and pores and that are covered with porous, fluffy little grains. Interestingly, the presence of large “spilled” structures is visible. The C4-750 sample (Figure A15) is characterised by an almost indistinguishable morphology; nevertheless, the surface is smoother and less porous than that of previous samples annealed at 750 °C. The NC-doped composition after annealing at 350 °C (Figure A8) presents a strongly porous structure that is full of irregular sintered particles that are usually characterised by a flat geometry. The little grains that cover the whole sample present a wide spread of morphology—irregular, hemispherical and spindle-like. After annealing at 550 °C (Figure A12), different from the previous sample, we cannot point out the presence of large, multi-walled sintered particles. The surface is composed mostly of agglomerates composed of granular and spherical grains with surfaces that are highly porous and full of cracks. Finally, the C3-1NC-750 sample (Figure A16) presents a morphology that is very similar to that of previous samples—it is composed of sizeable sintered structures with the presence of a few pores and cracks.

**Figure A21:**
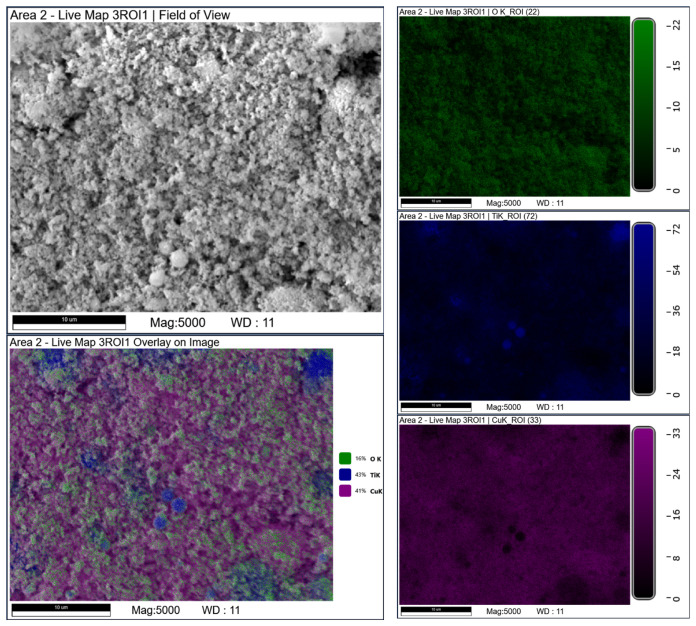
SEM-EDS images for C2 composition annealed at 350 °C.

**Figure A22:**
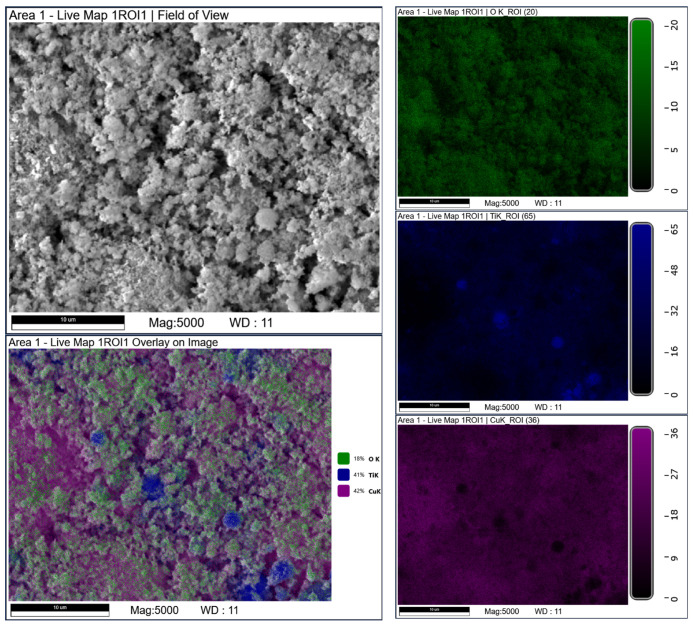
SEM-EDS images for C3 composition annealed at 350 °C.

**Figure A23:**
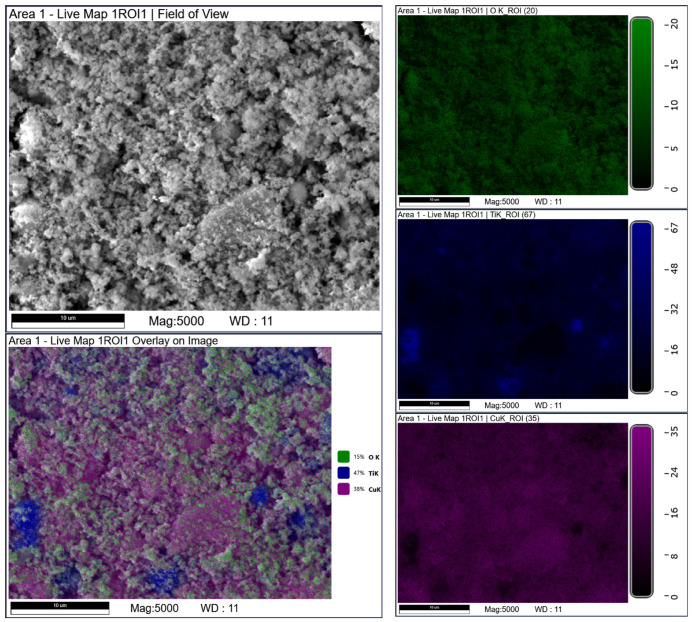
SEM-EDS images for C4 composition annealed at 350 °C.

**Figure A24:**
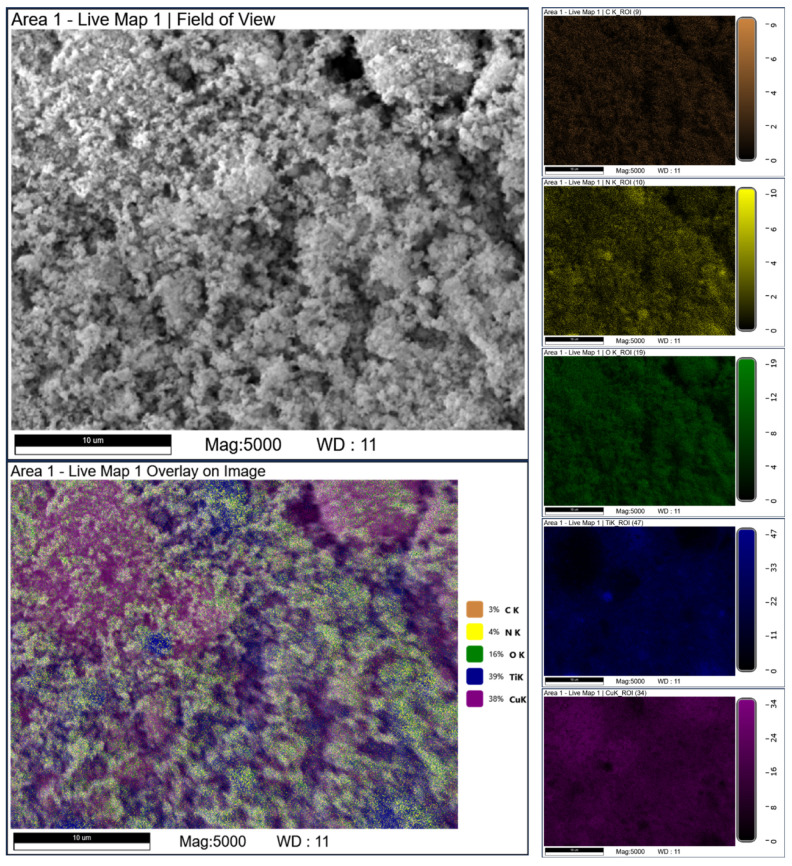
SEM-EDS images for C3-1NC composition annealed at 350 °C.

**Figure A25:**
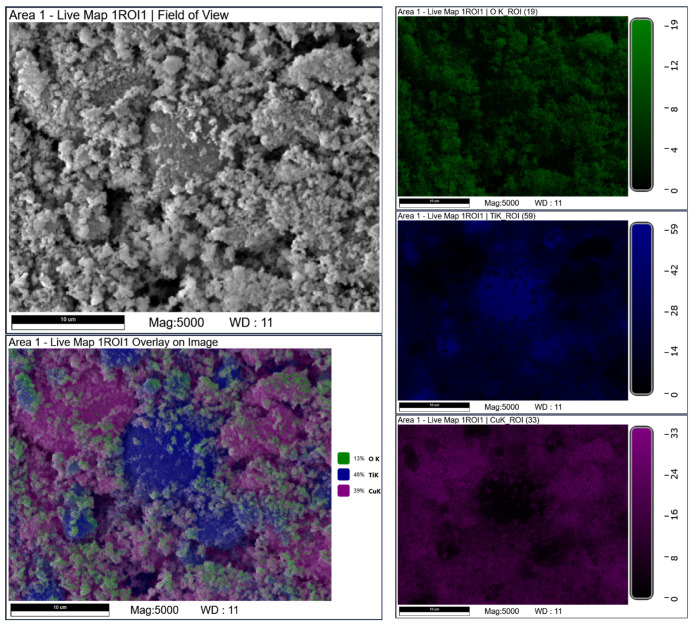
SEM-EDS images for C2 composition annealed at 550 °C.

**Figure A26:**
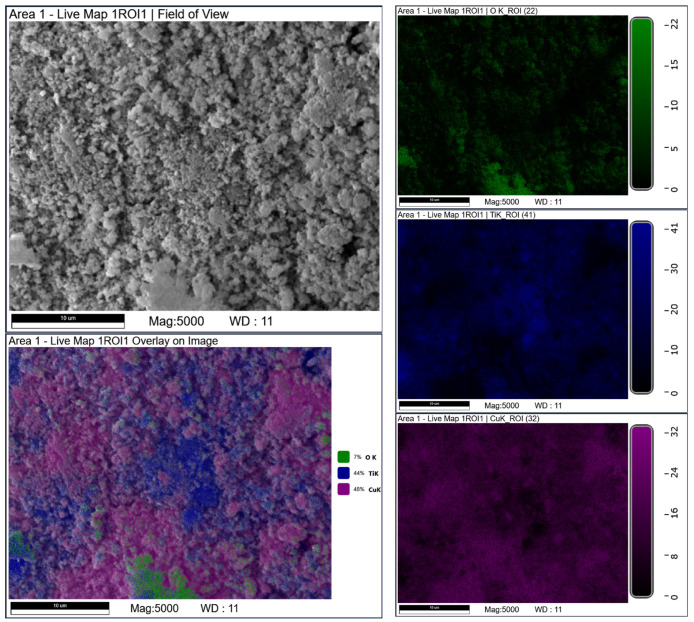
SEM-EDS images for C3 composition annealed at 550 °C.

**Figure A27:**
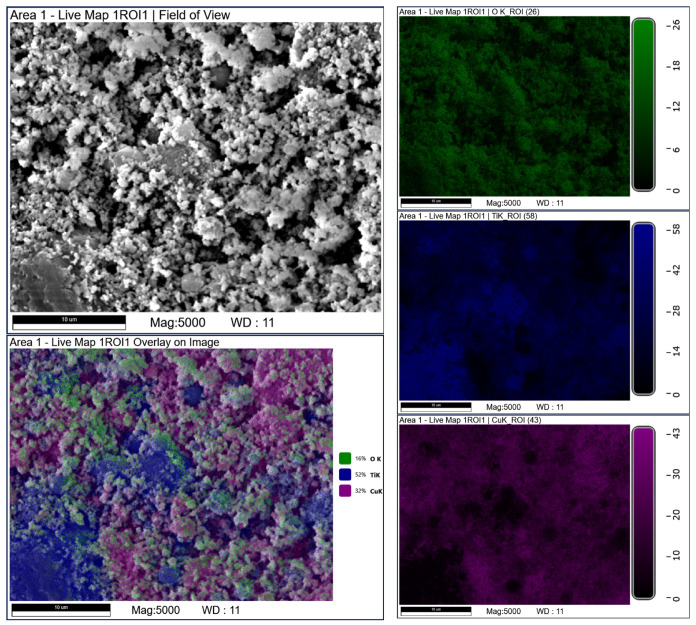
SEM-EDS images for C4 composition annealed at 550 °C.

**Figure A28:**
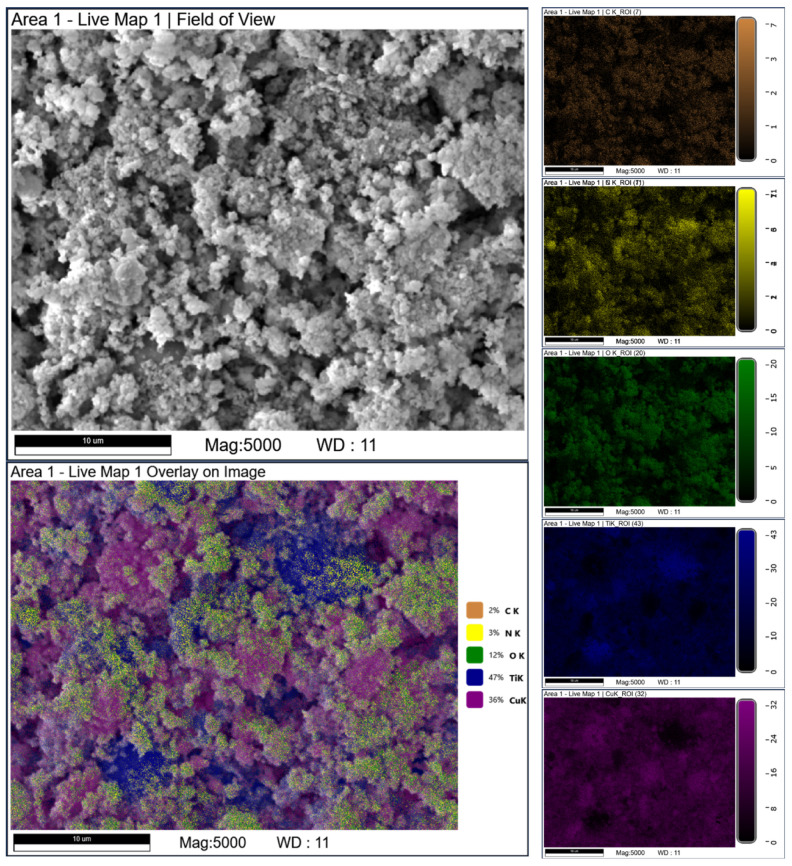
SEM-EDS images for C3-1NC composition annealed at 550 °C.

**Figure A29:**
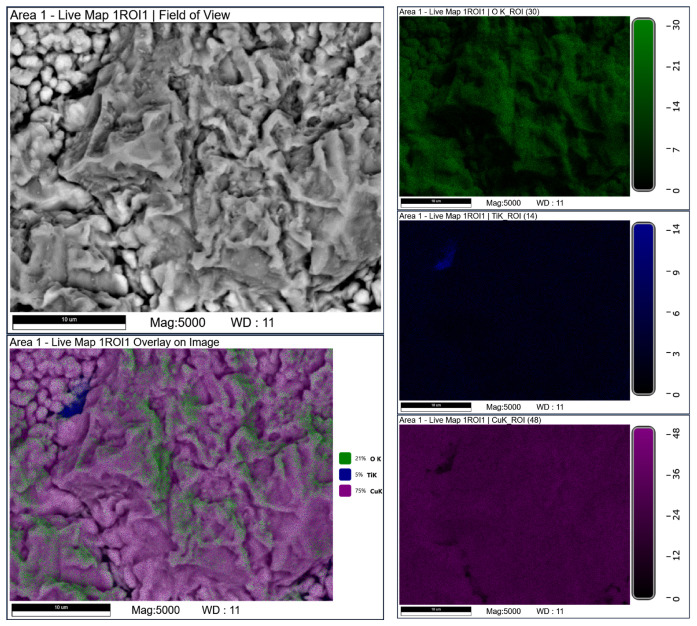
SEM-EDS images for C2 composition annealed at 750 °C.

**Figure A30:**
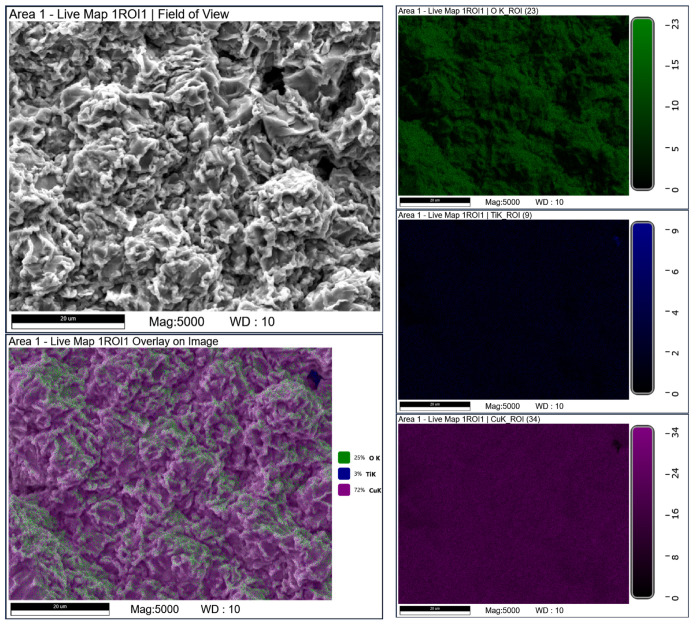
SEM-EDS images for C3 composition annealed at 750 °C.

The results of the SEM-EDS tests are presented in Figure A21, Figure A22, Figure A23, Figure A24, Figure A25, Figure A26, Figure A27, Figure A28, Figure A29 and Figure A30. SEM-EDS study of the C2-350 sample shows a quite equal distribution of Cu atoms except for places with increased content of Ti, which are significantly less homogeneously distributed. The areas with higher Ti content are both single micrometric fuel grains and agglomerates composed of nano-Ti. The distribution of oxygen is even in all samples. Analysis of the C2-550 samples indicates that the observed sintered, multi-walled particles are composed mostly of Ti and O atoms—most likely titanium oxides. At the same time, the small particles are composed mostly of Cu and O atoms. For C2 samples annealed at 750 °C, the occurrence of Ti atoms is less pronounced on the surface of the samples than in the case of previous samples. There are only few areas that are rich in Ti atoms (multi-walled particles) and they are covered with quite evenly distributed Cu and O atoms.

The C3-350 samples exhibit atom distributions that are nigh-identical to C2-350—the presence of Cu and O atoms is even in the sample, whereas for Ti, areas with increased content and exclusion of Ti and Cu atoms are present. After annealing of the C3 composition at 550 °C, the Ti distribution is limited to the presence of sintered, multi-walled particles composed of Ti and O. The distribution of oxygen atoms is quite even, whereas the Cu atoms are present in areas depleted of Ti. The C3-750 sample presents an even distribution of Cu (while maintaining an exclusive effect with Ti atoms). However, we can also notice low Ti content and areas full of Cu atoms that at the same time are depleted of O atoms. The Ti atoms are concentrated only in one visible spot.

The C4-350 sample presents a slightly different arrangement of atoms. The Ti atoms are poorly distributed among the sample and formed large agglomerates of nano-Ti. The exclusion between Ti and Cu is still visible, while O atoms are distributed equally. After annealing at 550 °C, the distribution is more similar to previous samples—the sintered particles are composed of Ti and O atoms and the oxygen atom distribution is even in whole sample, whereas the areas that are rich in Cu atoms do not contain Ti atoms. The C4-750 sample is characterised by the presence of evenly distributed Cu atoms, low content of equally distributed Ti and the occurrence of areas without O atoms.

**Figure 2:**
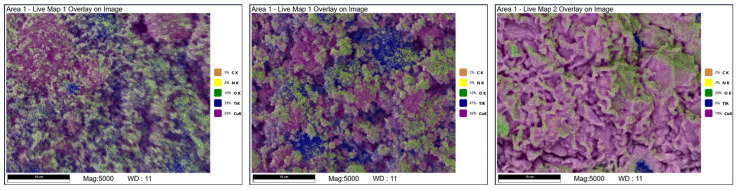
SEM-EDS images of the C3-1NC sample annealed at 350 °C (on the **left**), 550 °C (in the **centre**) and 750 °C (on the **right**).

The C3-1NC-350 composition is characterised by an even distribution of C, N and O atoms on the whole surface (Figure 2). The distribution of titanium atoms is clearly superior than for compositions without the NC additive—however, places with slightly increased Ti content can be found. At the same time, these areas are depleted of Cu atoms. After annealing at 550 °C, the C, N and O atoms are still distributed equally. It is interesting that the Ti atoms are distributed over the whole surface, with some areas having higher Ti content—namely, the previously described large sintered particles (composed of Ti and O). The Cu atoms are distributed quite equally and are present in small particles with varied morphologies. The EDS tests of the C3-1NC-750 sample showed similar dependencies as previous samples that were annealed at a temperature of 750 °C. Ti atoms are present only in a few areas, with copper atoms being excluded in these areas. The O atom distribution is characterised by the occurrence of areas that are deprived of that element.

To sum up, SEM and SEM-EDS analyses indicate the following reaction process. During the preliminary reaction at a low temperature (samples annealed at 350 °C), the agglomeration and oxidation of titanium takes place, with simultaneous oxidising agent decomposition. At a higher temperature (550 °C), after the main combustion reaction, multi-walled sintered TiO_2_ particles are present and are covered with smaller, fluffy and usually hemispherical or granular particles composed of Cu, CuO and Cu_2_O. At 750 °C, the whole sample surface is covered with remelted copper and residual oxidising agent, which explains the low signal for Ti atoms, which are linked as TiO_2_. At the same time, the analysis of the results points out the diffusion of oxygen and the oxidising agent into metallic fuel.

### 2.2. Raman Spectroscopy

**Figure 3:**
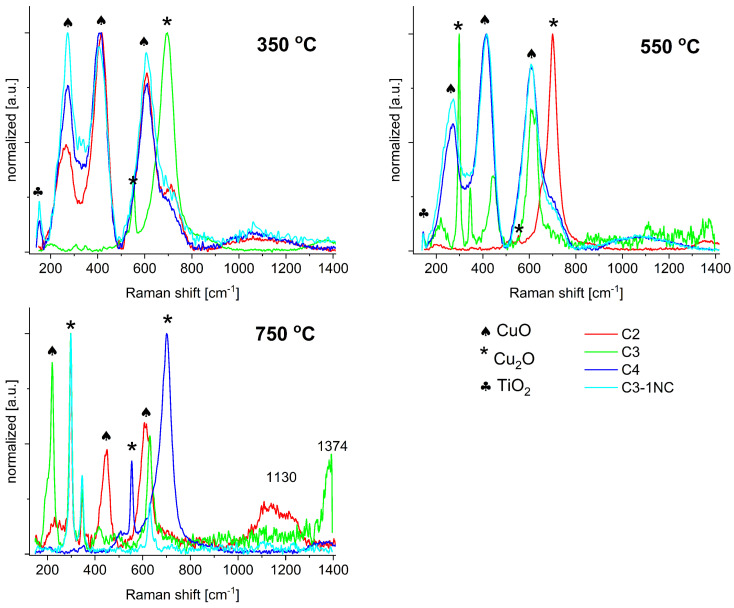
Raman spectra of samples annealed at different temperatures—350, 550 and 750 °C.

The Raman spectra of the NT samples after annealing at 350, 550 and 750 °C are collated in Figure 3.

The Raman spectrum of the annealed NT samples at a temperature of 350 °C corresponds to a mixture of Cu_2_O and CuO with only a small amount of TiO_2_. The signals at 217 and 415 cm^−1^ are attributed to Cu_2_O. Additionally, the spectra feature an active infrared mode of Cu_2_O located at 631 cm^−1^, which is indicative of a large number of structural defects in the Cu_2_O phase, as also reported in the literature [21]. The modes at 295, 434 and 639 cm^−1^ originate from CuO, as supported by reported data from the literature [22]. The presence of traces of TiO_2_ is confirmed by the signal at 141 cm^−1^, which is the highest-intensity signal of anatase [23]. In the spectra of the samples annealed at 750 °C, multi-phonon modes appear at 1130 and 1374 cm^−1^, originating from copper oxides, which suggests the presence of combustion products in the defective crystalline part. An important take-away here is that samples annealed at 350 °C can be considered well-ordered, whereas annealing at higher temperatures leads to an increasingly amorphous nature of the samples.

### 2.3. XRD Tests

**Figure A33:**
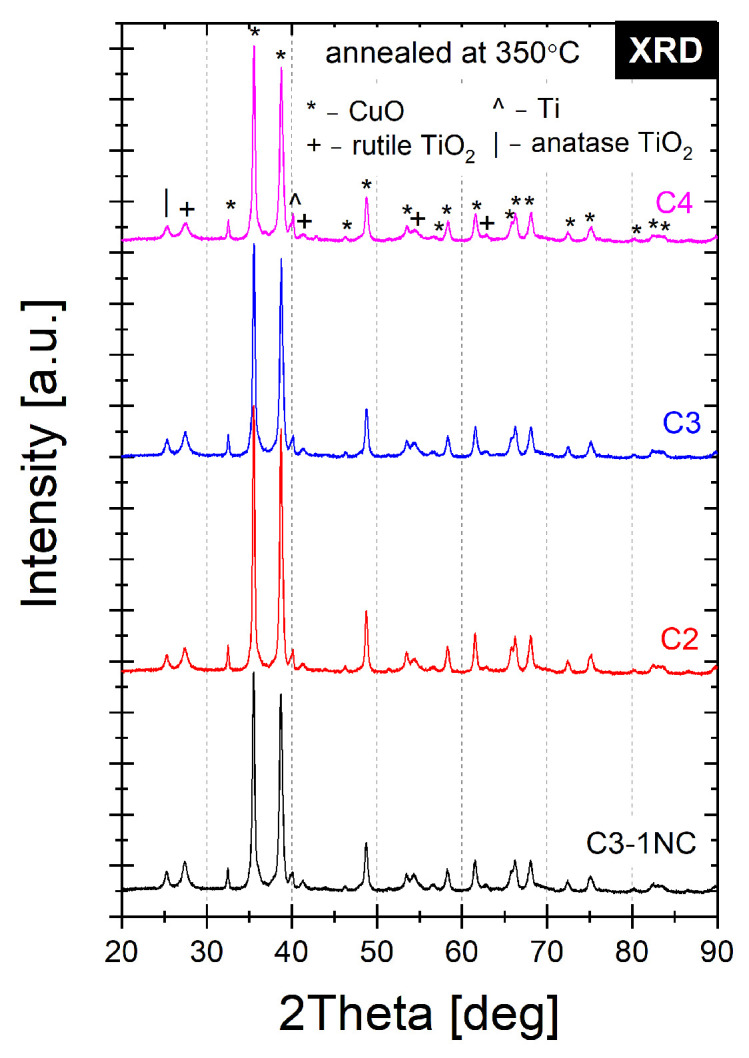
XRD patterns for the combustion annealed at 350 °C. The main crystalline phases have been marked for clarity.

**Figure A34:**
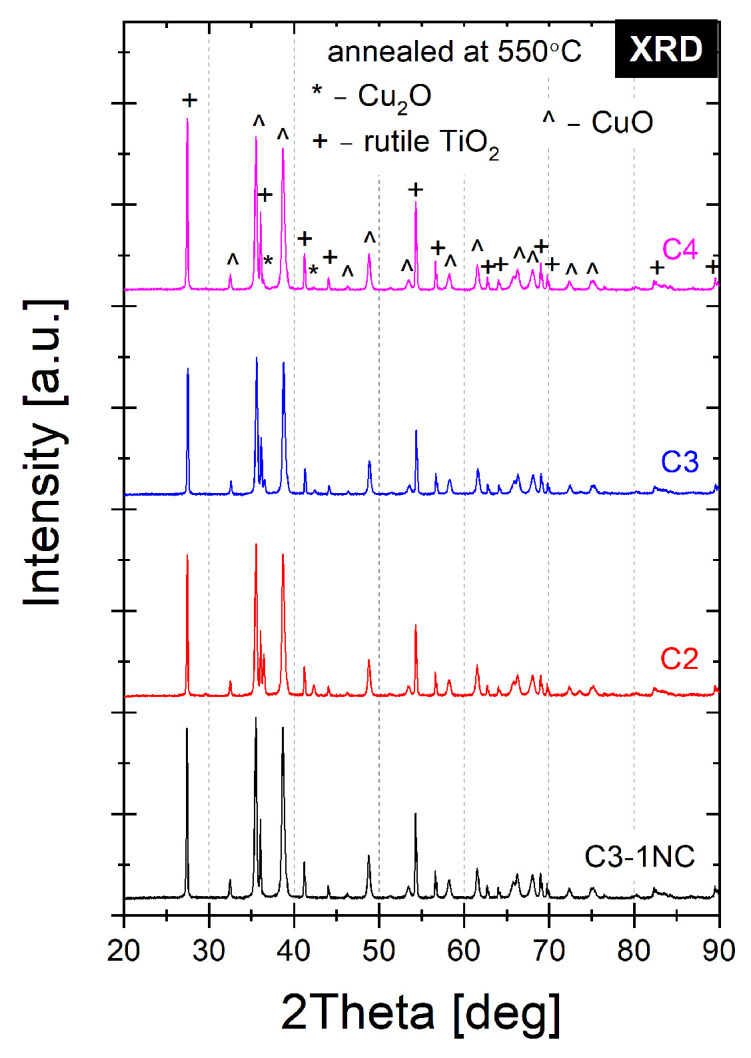
XRD patterns for the combustion annealed at 550 °C. The main crystalline phases have been marked for clarity.

**Figure A35:**
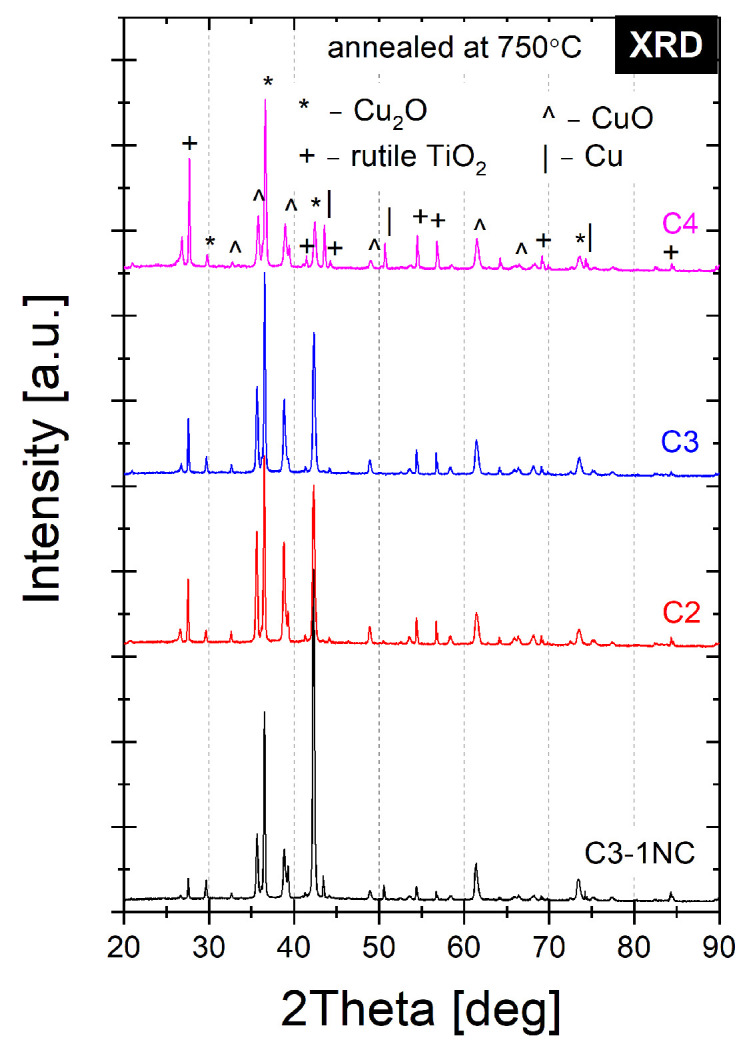
XRD patterns for the combustion annealed at 750 °C. The main crystalline phases have been marked for clarity.

The XRD patterns of the reactants after annealing at 350, 550 and 750 °C are illustrated in Figure A33, Figure A34 and Figure A35. The samples annealed at 350 °C are a multi-component system (Table 1) consisting of the tenorite CuO phase (PDF Card No. 01-080-1268), the rutile TiO_2_ phase (PDF Card No. 03-065-1118), the anatase TiO_2_ phase (PDF Card No. 01-075-2547) and the hexagonal Ti phase (PDF Card No. 01-089-2959). The samples annealed at 550 °C indicate a transition of the Ti and anatase phases into the most stable phase, which is rutile. Additionally, the phase of the CuO system is revealed. In the case of samples annealed at 750 °C, the Cu (PDF Card No. 03-065-9743) and the cuprite Cu_2_O (PDF Card No. 01-078-2076) phases are also identified.

### 2.4. DSC/TG Tests

At the beginning of the measurements, a small decrease in the mass of the tested samples is observed, which is connected with the evaporation of adsorbed water and residual solvent (originating from the composition preparation process) from the surface of the samples. In the range of 150–350 °C, a 0.5–1.0 wt.% mass loss is observed and is accompanied by a minor exothermic reaction, except for the C3-1NC composition. For C3-1NC, a distinct exothermic peak is observed around 190 °C, along with higher mass loss—up to 2 wt.%—which stems from NC decomposition. For all compositions, the mass loss may be ascribed to a preliminary reaction between the components, occurring with the release of gaseous oxygen from the system. The released oxygen is likely the oxygen dissolved in Ti that is being released due to the ongoing oxidation of titanium. The dependence of the shape of the DSC and TG curves on the equivalence ratio of the compositions annealed at 350 °C points to the presence of slow reactions that take place mostly in the solid phase.

**Figure 4:**
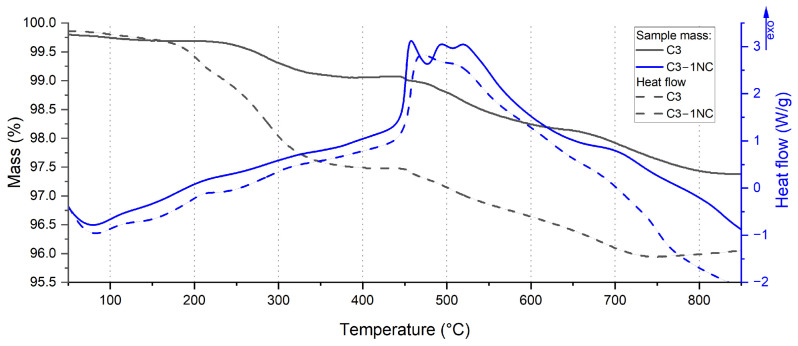
Example DSC/TG curves for C3 and C3-1NC compositions for heating rate of 20 K/min over limited range of heating temperatures.

Above 350 °C, both the heat flow and mass loss curves are stable up to a temperature of 440–460 °C, at which point the main combustion reaction starts. The ignition temperature is similar for all tested samples. Above this temperature, rapid mass loss takes place, up to 600 °C, with a short stabilisation above this temperature. At the same time, the main combustion reaction manifests a double exothermic peak in the thermogram, which is likely derived from the presence of a limiting stage during the reaction. A single, smooth peak is visible only for the C3-1NC composition (Figure 4).

Above 600 °C, the third exothermic peak can be observed and may be attributed to a post-combustion reaction between the residual components and the intermediate products. For the C2 composition, we also notice a mass decrease above this temperature resulting from residual CuO decomposition. For part of the measurements, one can notice an increase in the mass above 700–800 °C. The observed effect is observed only for low heating rates and stems from the reaction of combustion residues with air flowing into the chamber as a result of reverse gas currents.

**Figure 5:**
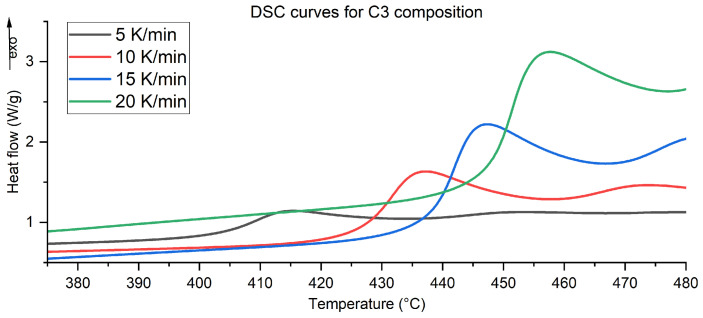
Example DSC/TG curves for C3 composition for different heating rates.

In almost all cases, an increase in the heating rate during the tests results in an increase of the total heat of the reaction (Table A1). The difference between the ignition temperatures was clear for all samples (Figure 5). The C3 sample exhibited the highest total heat of the reaction: twice as high as that of the C2 sample. It is interesting that there is a rapid increase in the total heat generated during the C4 composition analysis with an increase in the heating rate from 10 K/min to 15 K/min. The differences in the total heats arise from different values of ϕ for the compositions and, therefore, macroscopic factors like the contact between the fuel and the oxidising agent.

For the different heating rates, changes in the contribution of the subsequent reactions to the total reaction heat as a function of the composition and the heating rate were observed (Figure A40). For the C2 composition, with a higher heating rate, the participation of the second reaction dipped down almost by half, while the first reaction dominated for all heating rates. For the C3 composition, for the lowest heating rate (5 K/min), the contribution of the second reaction was above 55%, while for the heating rate of 20 K/min, it fell to approximately 20.5%. For the C4 composition, the situation is similar to that of the C2 composition, with almost a three-fold difference between the 5 K/min and 20 K/min heating rates and constant domination of the first reaction’s contribution. The C3-1NC composition is also characterised by a high contribution of the last reaction for low heating rates; moreover, the decrease in the contribution of this reaction is the highest among all tested samples (almost six-fold). At the same time, the contribution of the NC decomposition reaction increases with the heating rate, which points to more violent decomposition of NC and results in a higher mass of NT in this reaction.

With an increasing heating rate, the mass loss decreases in a linear fashion for most of the tested samples (Figure A41). A particularly significant difference is seen for the process taking place over the temperature range of 400–600 °C (main combustion reaction). Most likely, the mass loss is related to the evolution of gaseous oxygen from the reaction system, which is caused by the release of dissolved O_2_ from Ti nanopowder during its oxidation as well as by CuO decomposition. For a more violent reaction, which is in line with the heating rates, the reaction timescale is shorter; thus, less oxygen can escape the system.

The calculated energies of activation for the subsequent reactions are presented in the table below (Table 2).

The activation energy of the main combustion reaction decreases with an increase in ϕ (higher availability of the highly reactive fuel), with a significantly stronger decrease being observed upon the transition from C3 to C4. The addition of the NC binder leads to an increase in activation energy, which may result from the presence of solid residues from NC decomposition on the fuel and the surface of the oxidising agent. Moreover, the gas generating effect of NC decomposition may lead to larger mass diffusion lengths. For the last exothermic reaction, the trend is not linear. The transfer from the C2 to the C3 composition showed a lower E_a_ value, with a further increase for the C4 composition. Nevertheless, the changes in the calculated values between the compositions are little. The NC-doped composition is characterised by an approximately two-fold lower E_a_ for the last reaction. Most likely, the sintering-preventing effect of NC caused a lower conversion rate in the previous reaction (which may be confirmed by lower reaction heats in comparison to the C3 composition). Thus, during the last reaction, the availability of unreacted fuel is higher.

## 3. Discussion

Based on the performed tests, the following combustion mechanism appears plausible. The initial system is composed of CuO, Ti and TiO_2_ in the form of anatase. TiO_2_ is present as an oxide layer on the Ti grains, which limits access to the reactive fuel. During heating, the preliminary combustion reaction between Ti and CuO takes place up to temperatures of 350 °C. At the same time, the agglomeration of Ti particles and CuO decomposition take place, as confirmed by the occurrence of mass loss (DSC/TG) and a change in the sample morphology (SEM-EDS).

In the case of NC-doped compositions, the sintering and agglomeration process is significantly limited by gases generated during NC decomposition, whereas a high heat of reaction (in comparison with pure NT for temperatures below 250 °C) results from NC decomposition.

The main combustion reaction starts around 420–450 °C and leads to products composed of multi-walled sintered TiO_2_ particles covered with smaller granular and spherical CuO and Cu particles. The low ignition temperature indicates two important conclusions. Firstly, the reaction starts in the solid–solid phase and runs in both solid–solid and solid–liquid phase systems due to the high melting temperatures of the components (1668 °C for titanium and 1085 °C for copper [24,25]). Secondly, the low ignition temperature may stem from not only the high reactivity of nanometric titanium but also from the catalytic effect of Ti oxides on oxidising agent decomposition [16,26], whereas the decomposition temperature of nanometric CuO is equal to ∼530 °C [27].

The main combustion reaction manifests as a double peak in the DSC curves, except for the case of NC-doped NTs. This indicates that diffusion of the oxidising agent into the fuel core acts as a rate-limiting stage. During fuel oxidation, the increasing thickness of the Ti oxide layer hinders further diffusion of oxygen into the reactive core. Eventually, however, thermal expansion and changes in the chemical composition of the Ti oxides result in fracturing the oxide shell, leading into rapid movement of the oxidising agent deep into the reactive fuel core. The mentioned sealing/unsealing process of the reactive fuel core is related to the multi-step oxidation of Ti through lower oxides up to the stable TiO_2_. It is important to note that the intermediate oxides are characterised by different melting points and densities (Table 3).

The lack of this effect for the NC-doped compositions stems from lower agglomeration and sintering of the system during the previous reaction, which leads to higher intermediate contact between the components and a higher surface area of the components and, thus, shorter diffusion lengths and better availability of the fuel.

The direction of mass transfer into the fuel core is also confirmed by changes in the mass loss for different heating rates during the tests. A higher heating rate led to significantly lower mass losses, as the mass loss process is related to oxygen escaping from the system during CuO decomposition. In other words, the most violent reaction has a shorter time scale that does not facilitate the escape of oxygen. Additionally, the presence of areas with elevated content of Ti confirms diffusion deep into fuel grains; otherwise, the distribution of Ti atoms would be even throughout the whole system.

The results of the XRD tests indicate a combustion timescale that is sufficient for full oxidation of the Ti grains. Titanium spontaneously covers itself with a durable and tight layer of titanium oxides. Both titanium and anatase when annealed at a temperature of 550 °C indicated a transition to the most thermodynamically stable phase, which is rutile. Anatase is a metastable form and the process of its transformation into rutile is an irreversible reaction that takes place in the temperature range of 400–800 °C, which was confirmed by the XRD patterns. In the case of annealing at temperatures of 350 °C and 550 °C, the CuO phase was revealed, which proves the complete reaction of the copper particles present in the analysed compositions. The components of samples annealed at 750 °C of TiO_2_, Cu_2_O and CuO point to limited participation of the oxidising agent in the reaction. This was confirmed in the XRD patterns by the presence of Cu_2_O and Cu phases, which resulted from a deficiency in the oxygen needed for the complete oxidation of the copper. Two types of processes may occur during crystallisation, i.e., the incorporation of admixtures into the structures of the molecules included in the composition or the formation of structural defects that accelerate the transformation process of individual phases. Moreover, the above results are influenced by macroscopic factors related to the contact between components and the morphology, which undergoes rapid changes during the reaction of sintering processes at higher temperatures, which is also clearly visible in the SEM and SEM-EDS photos.

The decrease of the activation energy of this reaction stems from higher fuel content and macroscopic factors like contact between the fuel and the oxidising agent, which may be optimal at higher values of ϕ. The rapid increase in the activation energy for NC-doped compositions may result from two main reasons: firstly the oxidising agent and fuel particles may be covered with solid residues from NC decomposition; secondly, the limited sintering of components due to the gas-generating effect of the NC may deteriorate the initial contact between components.

The last combustion reaction results in the covering of the whole surface with remelted copper with a small amount of CuO and Cu_2_O granular particles. The reaction is related to a massive unsealing of reactive Ti cores covered with oxide layers as an effect of thermal expansion leading to the presence of cracks that facilitate violent penetration of the reactive fuel bed by oxygen and by the oxidising agent. The observed effects on the heating rate and this reaction’s contribution to the total heat confirm this hypothesis. With an increase in the heating rate, the contribution of this reaction dipped down, which stems from the lower content of reactive Ti remaining in system after the more rapid and violent main combustion reaction.

The residual CuO undergoes decomposition and, along with the Cu from the combustion reaction, covers the whole surface of the sample during solidification, which points to the presence of gaseous or liquid copper in the reaction system. The E_a_ values for the compositions without the NC additive are quite constant, whereas for the C3-1NC composition, it decreases almost by half, which may result from a lower reaction rate than those of the other reactions—therefore, there is higher reactive fuel availability and lower sintering of the whole system.

The morphologies of products annealed at different temperatures are quite similar between samples for all conditions. Nevertheless, we may notice an enhanced level of sintering with increased fuel content in compositions and a strong influence of gases from NC decomposition on the morphology. Therefore, we may point out that the combustion mechanism of the Ti/CuO and Ti/CuO/NC systems is in line with a reactive sintering (RS) mechanism [30]. For the NC-doped composition, the reaction mechanism most likely is slightly different due to effect of the presence of the gas-generating NC. NC decomposition results in less sintering and the grouping of the reaction components into smaller clusters due to the presence of a strong pressure wave. For Ti/CuO systems, the heat transfer is based on conduction, while for Ti/CuO/NC, the large amount of gases enables a significant contribution of convective heat transfer. We can expect a reaction mechanism similar to the one described in [12], which proved the formation of reacting micro-clusters composed of micrometric nanothermite agglomerates that are “held” by hot gases for a time on the order of the timescale of full combustion.

In terms of the initial chemical reaction mechanism, the first reactions take place at or below 350 °C. At this stage, an initial solid phase reaction between *Ti* and *CuO* (Equations (1), (2) and (4)) and the evolution of oxygen being dissolved in *Ti* upon the oxidation of *Ti* (Equation (3)) are observed [31,32] For samples containing nitrocellulose, a third reaction, i.e., the decomposition of nitrocellulose, is also observed.

(1) $$Ti+2CuO\to TiO+Cu_{2}O$$

(2) $$Ti+2xCuO\to TiO_{x}+xCu_{2}O$$

(3) $$O_{2(Ti)}\to O_{2(g)}$$

(4) $$TiO+2CuO\to TiO_{2}+Cu_{2}O$$

*TiO*_2_ was observed by both Raman spectroscopy and XRD, with detection of both rutile and anatase indicating that partial oxidation of titanium took place in addition to the occurrence of the oxide shell on the surface of the Ti grains prior to the reaction. The *TiO* and *TiO*_x_, whose presence during the combustion reaction is hypothesised, cannot be detected by XRD if it is amorphous. Additionally, between annealing and measurement, the samples characterised by nanometric sizes could have been oxidised by air to the most stable product. Similarly, Cu_2_O was detected only by Raman spectroscopy, suggesting only a minor degree of conversion being achieved in the timescale of the experiment. The amount of dissolved oxygen that is released due to Ti oxidation corresponds to the degree of conversion and accounts for the observed mass loss of approximately 1 wt.%.

In the case of NC-containing samples, a much greater mass loss (approximately 2.5 wt.% for the C3-1NC sample) is observed. While part of this mass loss is accounted for by the 1 wt.% NC content undergoing a reaction to gaseous combustion products, the remaining mass loss is likely caused by the resulting convection promoting the release of oxygen dissolved in the *Ti*.

(5) $$TiOx+\left(2-x\right)CuO\to TiO_{2}+\left(1-0.5x\right)Cu_{2}O$$

(6) $$Ti+4CuO\to TiO_{2}+2Cu_{2}O$$

The next stage, i.e., the main stage of combustion, takes place at or below 550 °C and follows Equation (5); it is related to further oxidation of intermediate titanium oxides. Contrary to the case of 350 °C, the degree of conversion in this reaction is much higher, being nigh-100%, as for samples conditioned at this temperature, Ti is no longer observed by XRD. A similar mechanism was reported in the literature [17], where gradual oxidation to stable *TiO*_2_ was suggested. The reduction of the *TiO*_2_ layer to *TiO*_x_ and *TiO* during heating, which facilitates oxygen migration into the fuel core, was, in turn, postulated in another report on the subject [16]. Moreover, the double peak present in the DSC thermograms may be attributed to rapid oxidation of *TiO*_x_ to *TiO*_2_, which acts as a significantly stronger barrier to oxygen diffusion [16,17]. The subsequent cracking and unsealing of the *TiO*_2_ layer at higher temperatures facilitates a further combustion process.

Interestingly, the main combustion stage is followed by afterburning (most likely combustion of residual *TiO*_x_) and follow-up decomposition of the oxidising agent (Equations (7) and (8)). This is seen by the manifestation of metallic copper in the combustion products and the decrease in mass.

(7) $$2CuO\to Cu_{2}O+0.5O_{2}$$

(8) $$Cu_{2}O\to 2Cu+0.5O_{2}$$

The effect of the NC additive on the combustion process is mostly macroscopic and was discussed in detail in previous parts. However, the presence of gases from NC decomposition may lead to partial reduction of Ti oxides at the combustion front. On the contrary, the reaction of NC decomposition products (e.g., carbon black or nitrogen) with the reactive *Ti* core [33] can be neglected (Equations (9) and (10)). Nevertheless, due to the low content of NC in the tested composition (1 wt.%), the effect may be insignificant in terms of quantitative and qualitative analyses.

(9) Ti+C→TiC

(10) $$Ti+0.5N_{2}\to TiN$$

## 4. Materials and Methods

### 4.1. Materials

The components (Table 4) were used to produce compositions C2–C4 and C3-1NC Table 5. All components were used as received, without any purification.

### 4.2. Preparation of Compositions

The compositions were prepared for three different values of ϕ: 1.2, 1.4 and 1.6 for C2, C3 and C4, respectively. Additionally, the composition characterized by ϕ equal to 1.4 was prepared with a 1 wt.% NC additive to form the C3-1NC composition.

All compositions were prepared using the same procedure. Firstly, all components were weighed and dried for 12 h at a temperature of 60 °C. The solid NC was obtained via drying of collodium solution at a temperature of 90 °C for 12 h. After drying, the powders were transferred into glass bottles and filled with propan-2-ol in such a manner as to achieve solid loading equal to 125 mg/cm^3^. In the next step, each composition was mixed for 30 min with a magnetic stirrer, was sonicated for 30 min (UH50 sonicator with a 0.7/0.3 work/break cycle) and was stirred for another 48 h. The prepared suspensions were electrosprayed in small portions (1 cm^3^) with the following conditions: voltage difference equal to 19 kV, distance between the collector and nozzle equal to 10 cm, nozzle internal diameter equal to 0.50 mm and flow rate of 3 cm^3^/h. After the deposition process, the compositions were gently removed from the collectors with a spatula, were dried for 12 h at a temperature of 90 °C and were poured into sealed containers.

### 4.3. Preparation of Samples for SEM-EDS and XRD Tests

**Figure A36:**
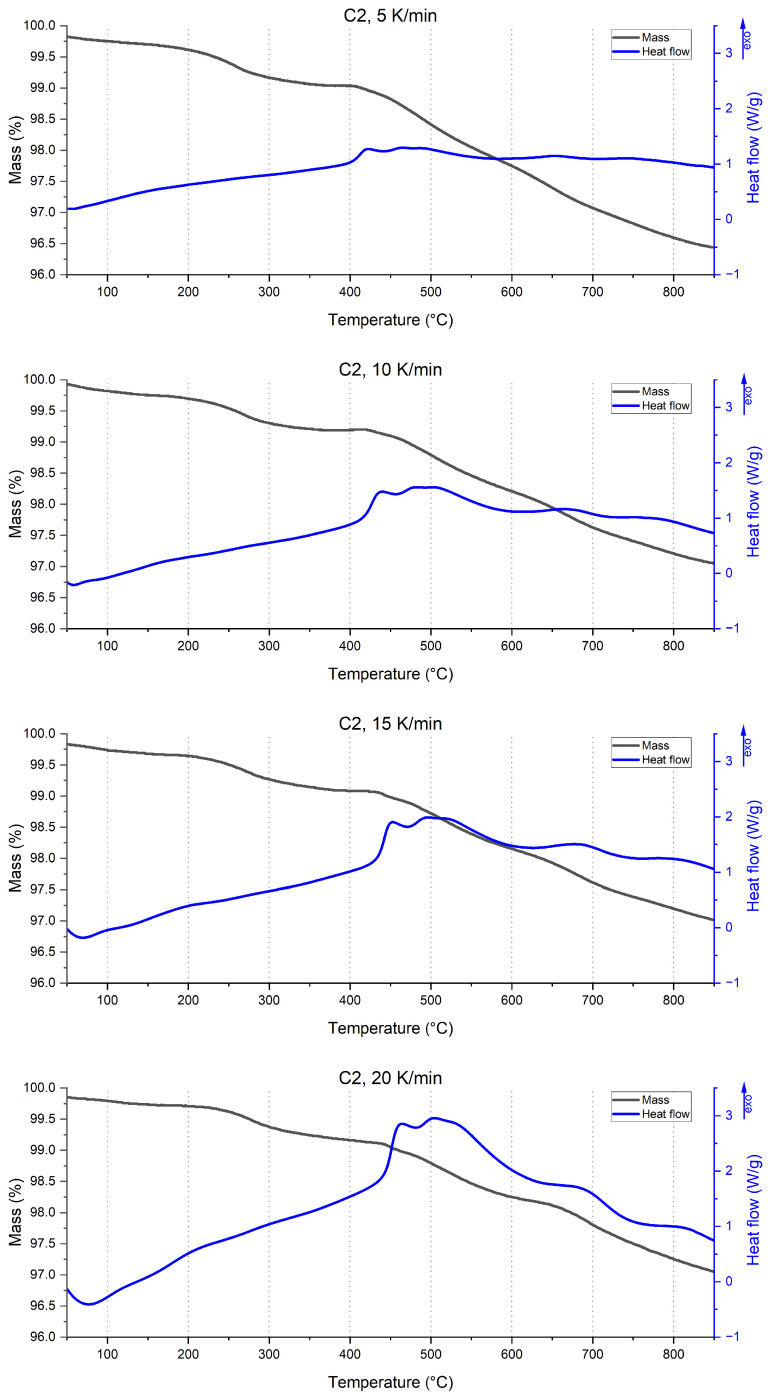
DSC/TG curves for C2 composition.

**Figure A37:**
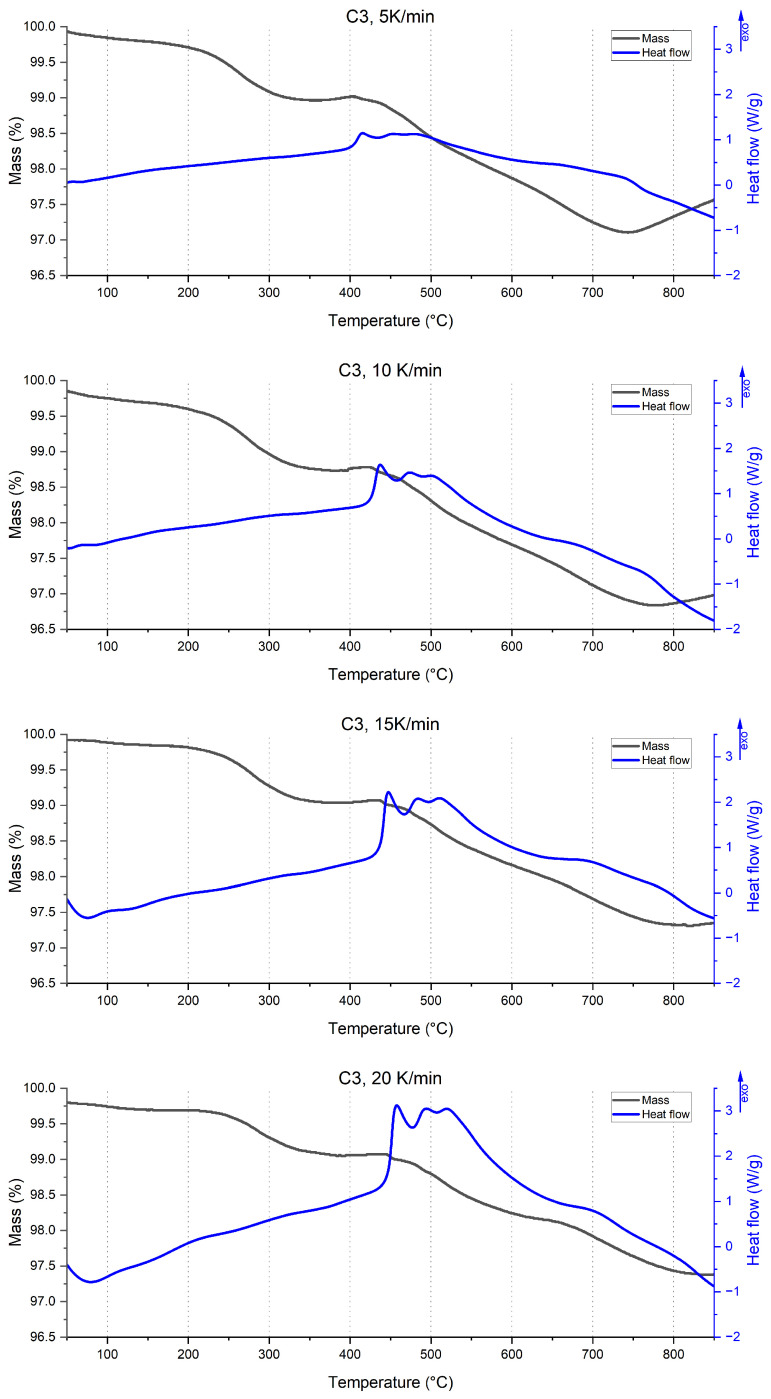
DSC/TG curves for C3 composition.

**Figure A38:**
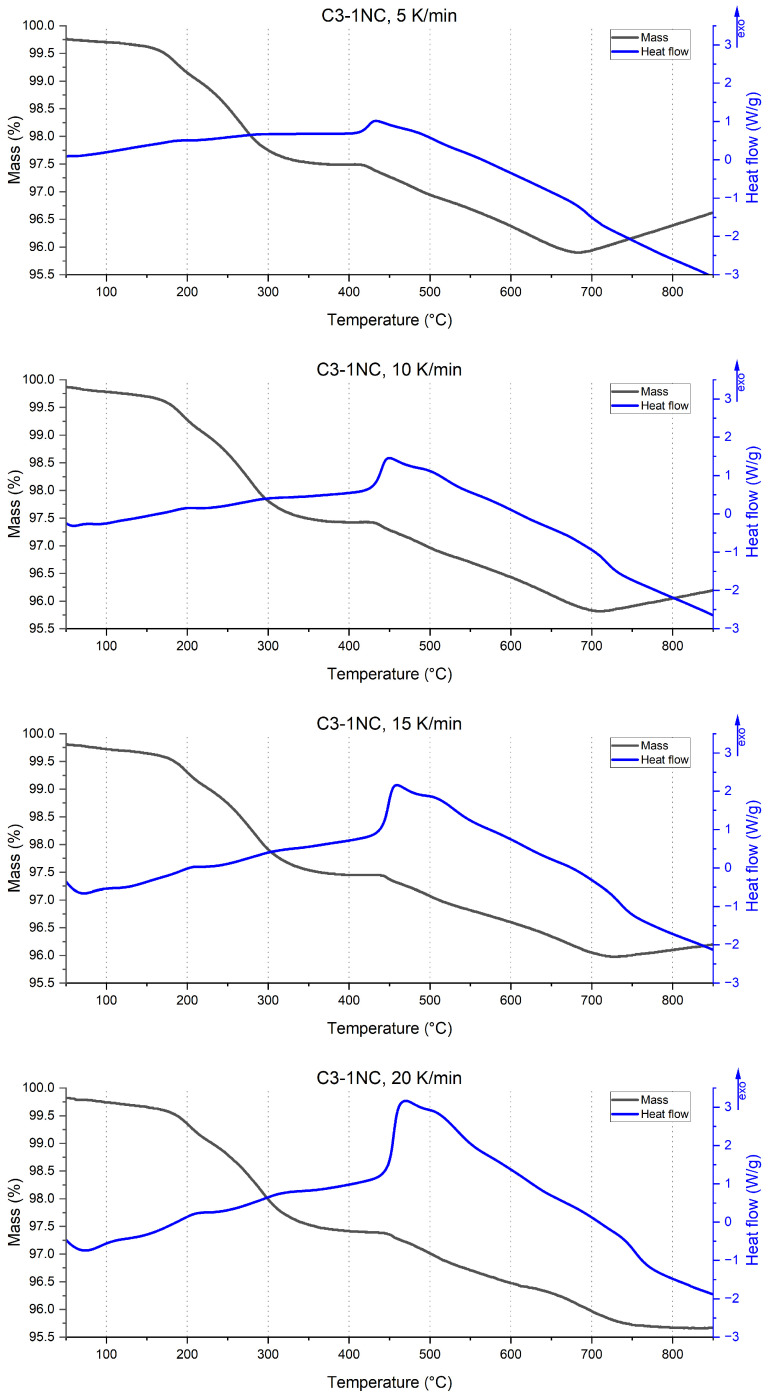
DSC/TG curves for C3-1NC composition.

**Figure A40:**
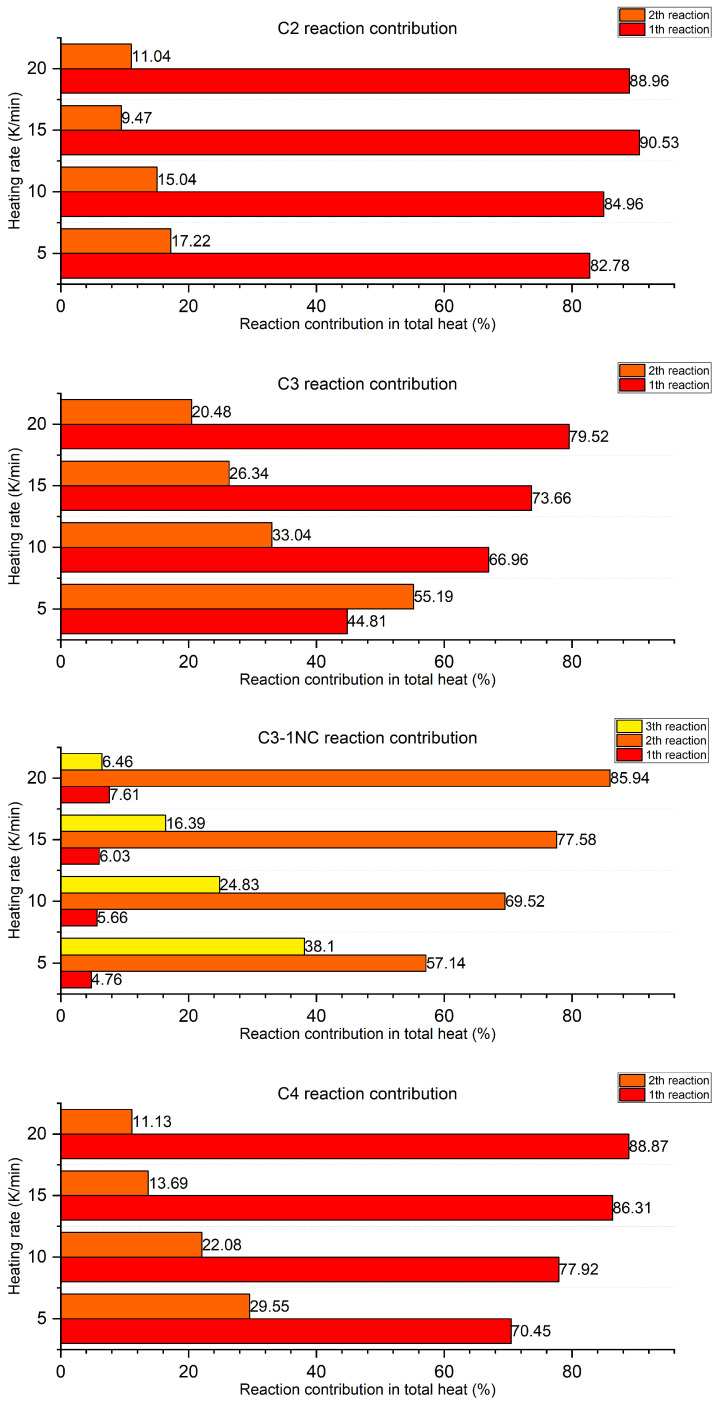
Contributions of the individually identified reactions to the total reaction heat for the tested NTs.

**Figure A41:**
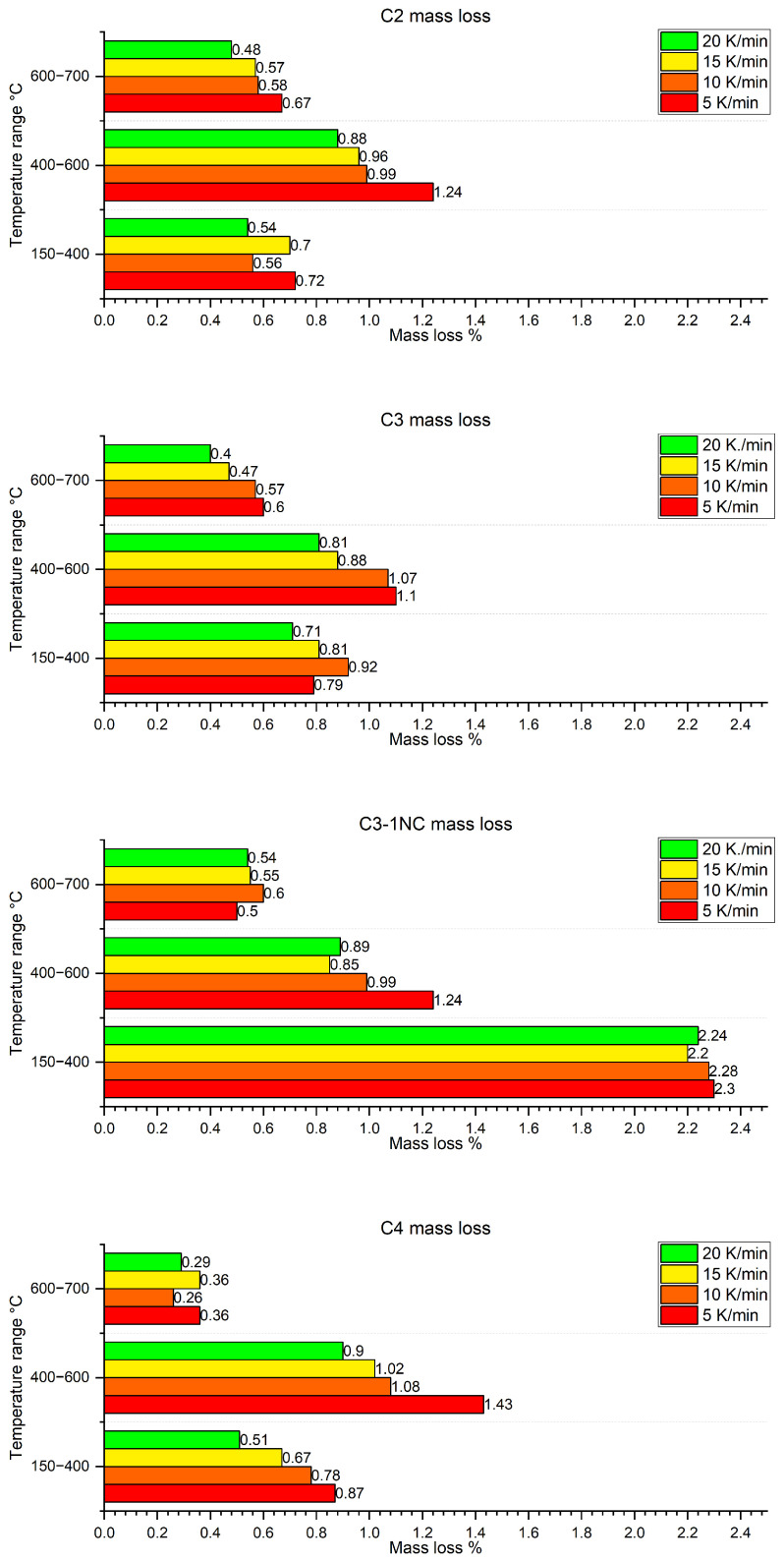
Mass loss of tested compositions during DSC/TG tests.

The compositions were thermally conditioned in an argon 5.0 atmosphere in an oven for 6 h. A heating rate of 20 K/min and a cooling rate of 0.5 K/min were used for all samples. Based on the results of the DSC/TG measurements (Figure A36, Figure A37 and Figure A38), the conditioning temperatures were selected:

- 350 °C—Below this temperature all samples showed significant mass loss, which we attributed to a preliminary reaction between Ti and CuO along with NC decomposition in the case of the C3-1NC composition. Hence, this temperature was chosen to verify that hypothesis.
- 550 °C—Below this temperature, the main combustion reaction took place, which is connected to significant mass loss and a high heat of reaction. Therefore, this temperature was chosen to assess the main combustion phase.
- 750 °C—Below this temperature, the last combustion reaction was observed, while mass remained fairly constant during this event. This temperature was chosen to verify the composition of the residues after all of the reactions.

All samples are described with labels appended with the conditioning temperature, i.e., C2-350 refers to composition C2 that was conditioned at 350 °C.

### 4.4. SEM-EDS Tests

The chemical compositions and morphologies of the thermally conditioned compositions were investigated using an FEI Inspect S50 (FEI, Hillsboro, OR, USA) scanning electron microscope (SEM) and an X-ray energy dispersive spectrometer with an EDS Octane Elect Plus detector and an EDAX Z2-i7 analyser (Bruker, Billerica, MS, USA), enabling the simultaneous acquisition of micrographs and energy dispersive X-ray (EDS) maps of the investigated samples. The working distance was equal to 10 mm, the acceleration voltages of the incident electrons were in range of 5–30 kV, the current intensity of the incident electronic beam was around 95 µA and the electronic beam spot size was equal to 3.

### 4.5. Raman Spectroscopy

Raman spectra were recorded with a Renishaw inVia Raman spectrometer and a 532 nm laser (Renishaw, diode laser, 380 mW at the source). The acquisition time for each scan and the number of accumulations were varied to improve the signal-to-noise ratio. Additionally, a large percentage (typically greater than 95 %) of the laser power was attenuated in order to prevent damage to the samples. The spectral resolution was ±1.2 cm^−1^. Spectra were typically collected using ×20 long-focus objectives (LEICA). Calibration of the spectrometer was carried out using a 520 cm^−1^ line of silicon. Data acquisition was carried out using the Renishaw WiRE 5.6 software package.

### 4.6. XRD Tests

X-ray diffraction measurements of the selected samples were performed on a Rigaku Miniflex 600 diffractometer using a D/teX Ultra silicon strip detector. The X-ray diffraction (XRD) patterns were recorded over a range of 20–90° 2θ using Cu-Kα radiation (λ = 1.5406 $\dot{A}$) operated at 40 mA and 15 kV using Bragg–Brentano geometry with a scanning step size of 0.01°. The exposure time at each point was 1.67 s without sample rotation. For all scans, the IHS slit = 5 mm, Soller slits = 2.5° and DS slit = 1.25° were used.

### 4.7. DSC/TG Tests

DSC/TG was performed with a TA Instruments SDT Q600. The argon 5.0 flow was equal to 200 cm^3^/min, the sample masses ranged from 9 to 10 mg, and the heating rates were 5, 10, 15 and 20 K/min. Ceramic crucibles closed with gold disc (with a 0.3 mm hole in the centre) were used in all tests.

### 4.8. Kinetic Analysis

The Kissinger and Ozawa methods were applied to the DSC curves to calculate the energy of activation [34]. The rate of reaction can be described by *k*(*T*) and *α*(*T*) functions and can be written as:

(11) $$\frac{d\alpha }{dt}=k\left(T\right)\alpha \left(T\right)$$

where *dα*/*dT* is the conversion rate, α is the conversion and *k*(*T*) is a temperature-dependent rate constant. The function of the conversion can be described as:

(12) $$f\left(\alpha \right)={\left(1-\alpha \right)}^{n}$$

where:

*n*—order of the reaction. The Arrhenius equation is described as:

(13) $$k\left(T\right)=Aexp\frac{-E_{a}}{RT}$$

where

A—pre-exponential factor,
*R*—gas constant,
*E_a_*—energy of activation.

Comparison of the equations leads to:

(14) $$\frac{d\alpha }{dt}={\left(1-\alpha \right)}^{n}*Aexp\frac{-E_{a}}{RT}$$

#### 4.8.1. Kissinger Method

After appropriate transformations, which are described in detail in [35], it is possible to present Equation (15) as:

(15) $$\frac{E_{a}\beta }{R{T^{2}}_{m}}={\left(1-\alpha \right)}^{n-1}*Anexp\frac{-E_{a}}{RT}$$

where:

β = *dT*/*dt*,
*T_m_*—maximum temperature peak for a given β.

The Kissinger method assumes that the β value does not affect (1 −α)^*n*−1^. So after logarithmic transformation of both sides, we have:

(16) $$ln\frac{\beta }{{T^{2}}_{m}}=ln\left(\frac{AR}{E_{2}}\right)-\left(\frac{E_{2}}{RT_{m}}\right)$$

The activation energy can be calculated from the slope of the line in the plot of *lnβ*/*T*^2^*_m_* vs. 1/*T_m_* by a k-multiple of the R constant.

#### 4.8.2. Ozawa Method

Equation (17) can be written for a linear β as:

(17) $$\int_{0}^{\alpha }\frac{d\alpha }{f(\alpha )}=\frac{A}{\beta }\int_{T_{0}}^{T}exp\left(\frac{-E_{a}}{RT}\right)dT$$

The above equation can be written as:

(18) $$logf\left(\alpha \right)=log\left(\frac{AE_{a}}{R}\right)-log\beta -2.315-0.4567\frac{E_{a}}{RT_{m}}$$

The activation energy can be calculated from the slope of the line (y = kx + b) in the plot logβ vs. 1/*T_m_* by k−/(−0.4567) multiplied by the R constant.

## 5. Conclusions

The combustion mechanism of the Ti/CuO system is in line with the RS mechanism and exhibits an unusual direction of mass transfer, i.e., into the fuel core, which is opposite of the trend observed for Al-based nanothermites. The NC-doped composition is characterized by a slightly different combustion mechanism originating from the fact that the evolution of hot gases interferes with the sintering process. The sintering process itself is clearly evidenced by changes in the sample morphology (SEM-EDS); moreover, it is more advanced for compositions with high Ti content (C4 composition).

The multi-step combustion process is limited mostly by macroscopic (e.g., homogeneity of the composition, contact between the fuel and oxidising agent and the equivalence ratio), mass and heat diffusion factors. A higher ϕ leads to a lower E_a_, higher reaction heats and a more violent combustion process. An interesting feature of the Ti/CuO system is the repeated sealing/unsealing of the reactive core of the Ti grains, which is caused by the formation of a titanium oxide shell on their surface, followed by cracking of this shell due to changes in the unit volume of the oxides (caused by changes in the chemical composition of the oxide shell).

The above effects is in line with the proposed reaction cycle, where the subsequent oxidation of Ti through TiO_x_ oxides explains the limited access of the oxidising agent to the reactive Ti core.

The tested systems, owing to their favourable properties, may find application both in civil and military industry. The tested systems can be used in primers and igniters, for the construction of NPED-type detonators, for microthrusters, for delay systems, in welding applications or as a base NT for EM-doped NSTEX systems or gas-generating systems, e.g., for ejection seats or airbags.

## Figures and Tables

**Table 1:** Content of annealed compositions.

Temperature of Annealing	Content
350	CuO, Ti, TiO_2_ (rutile), TiO_2_ (anatase)
550	CuO, Cu_2_O, TiO_2_ (rutile)
750	Cu, CuO, Cu_2_O, TiO_2_ (rutile)

**Table 2:** Calculated energies of activation. Values are expressed in kJ/mol.

Kissinger Method				
Reaction	C2	C3	C4	C3-1NC
1	-	-	-	87 ± 6
2	141 ± 28	134 ± 16	110 ± 37	157 ± 25
3	265 ± 32	253 ± 51	280 ± 43	129 ± 19
**Ozawa Method**				
Reaction	C2	C3	C4	C3-1NC
1	-	-	-	90 ± 6
2	145 ± 25	139 ± 15	116 ± 35	161 ± 24
3	266 ± 30	255 ± 48	275 ± 50	129 ± 19

**Table 3:** Melting points and densities of Ti and Ti oxides [24,28,29].

Compound	Melting Point (K)	Density (g/cm)^3^
Ti	1650	4.51
TiO	1750	4.94
Ti_2_O_3_	1839	4.49
Ti_3_O_5_	1774	4.42
TiO_2_	1843	4.23

**Table 4:** Materials used in composition preparation.

Component	Purity Grade/Concentration (wt.%)	Particle Size (nm)	Producer
Copper(II) oxide	wt. 99.99	40	Iolitec GmBH (Heilbronn, Germany)
Titanium	wt. 99.99	50	Iolitec GmBH (Heilbronn, Germany)
Propan-2-ol	99.9	-	Electro Chem (Warsaw, Poland)
Cellulose nitrate solution in 70/30 Et_2_O/EtOH	4–8	-	Sigma-Aldrich (Taufkirchen, Germany)

**Table 5:** Content of tested compositions.

Composition Name	Ti (wt.%)	CuO (wt.%)	NC (wt.%)
C2	26.5	73.5	-
C3	29.6	70.4	-
C4	32.5	67.5	-
C3-1NC	29.3	69.7	1.0

**Figure A31:**
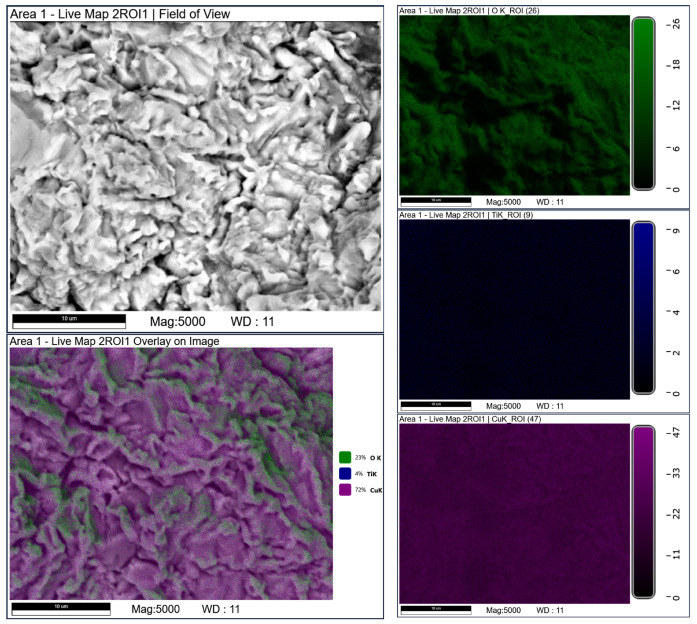
SEM-EDS images for C4 composition annealed at 750 °C.

**Figure A32:**
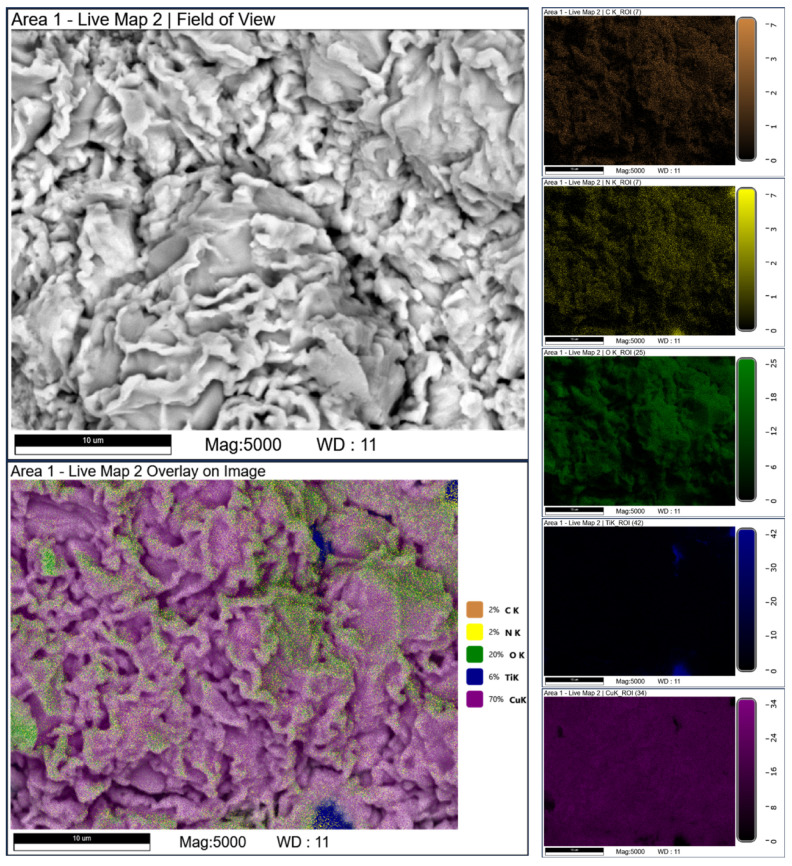
SEM-EDS images for C3-1NC composition annealed at 750 °C.

**Figure A39:**
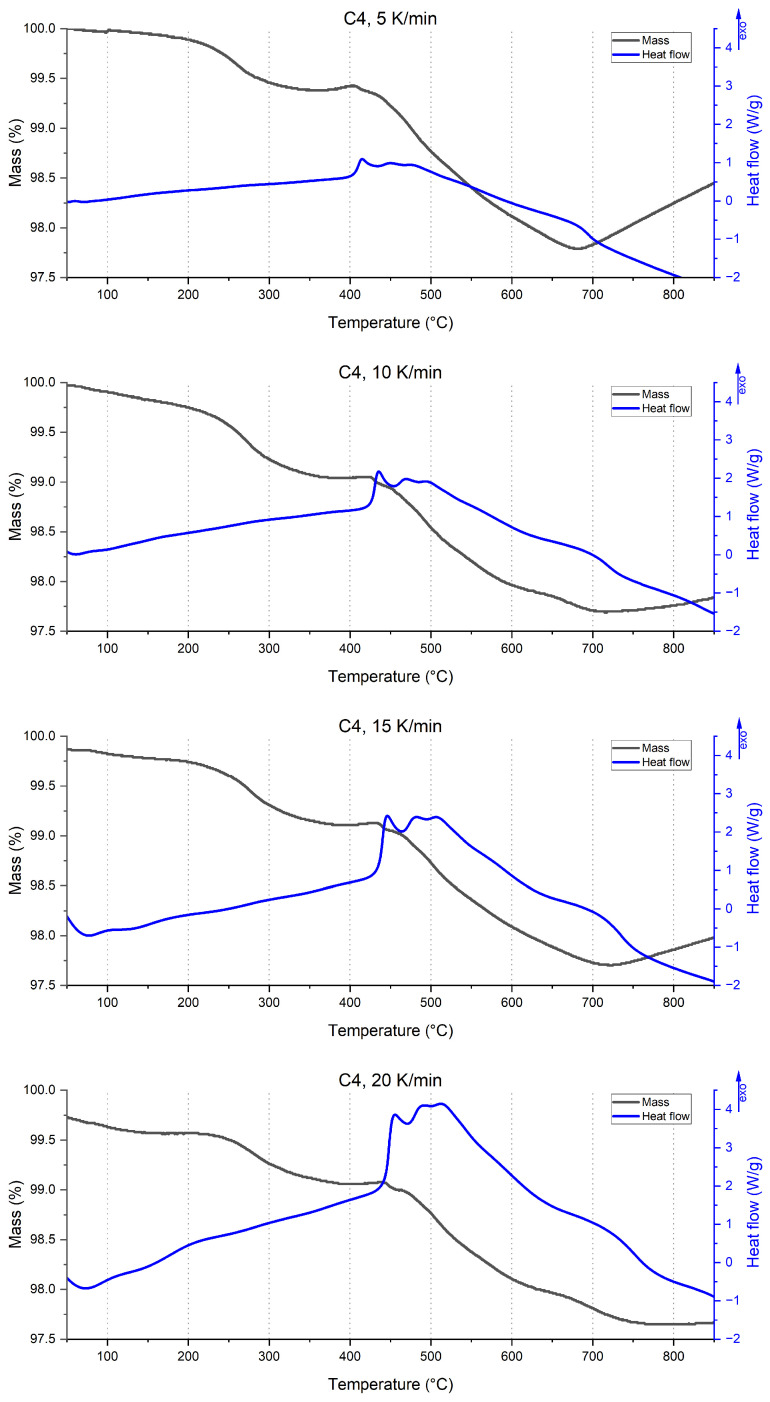
DSC/TG curves for C4 composition.

## Data Availability

The data presented in this study are available on request from the authors.

## Abbreviations

The following abbreviations are used in this manuscript:

NT	Nanothermite
NSTEX	Nanostructured thermite and explosives
NPED	No primary explosive device

## Appendix A

### Appendix A.3. DSC/TG Tests

**Table A1:** Heat of reactions of tested compositions.

C2 composition				
Heating rate	5 K/min	10 K/min	15 K/min	20 K/min
1st reaction	-	-	-	-
2nd reaction	149	192	239	282
3rd reaction	31	33	25	35
Sum	180	226	264	317
C3 composition				
Heating rate	5 K/min	10 K/min	15 K/min	20 K/min
1st reaction	-	-	-	-
2nd reaction	207	302	400	427
3rd reaction	255	150	142	110
Sum	462	451	543	537
C4 composition				
Heating rate	5 K/min	10 K/min	15 K/min	20 K/min
1st reaction	-	-	-	-
2nd reaction	248	307	473	463
3rd reaction	105	87	75	58
Sum	352	394	548	521
C3-1NC composition				
Heating rate	5 K/min	10 K/min	15 K/min	20 K/min
1st reaction	15	20	24	34
2nd reaction	183	252	303	386
3rd reaction	122	90	64	29
Sum	320	362	391	449
